# MALDI-TOF-MS based identification and molecular characterization of food associated methicillin-resistant *Staphylococcus aureus*

**DOI:** 10.1038/s41598-017-11597-z

**Published:** 2017-09-12

**Authors:** H. M. Manukumar, S. Umesha

**Affiliations:** Department of Studies in Biotechnology, University of Mysore, Manasagangotri, Mysuru-570006 Karnataka India

## Abstract

Food-borne methicillin resistant *Staphylococcus aureus* (MRSA) is involved in two-fold higher mortality rate compared to methicillin susceptible *S*. *aureus* (MSSA). Eventhough Mysuru recognized as cleanest city in the world, prevalence of food contamination is not detailed. The aim is to screen food samples from Mysuru area and to characterize MRSA strain, employing MALDI-Biotyper, multiplex PCR to distinguish between MRSA and MSSA by PCR-coupled single strand conformation polymorphism (PCR-SSCP). Of all the food-borne pathogens, *S*. *aureus* contamination accounts for 94.37 ± 0.02% (*P* < 0.01), strains characterized by means of *nuc* genes, followed by species specific identification by *Coa*, *Eap* and *SpA* genes and multiplex PCR to confirm the presence of three methicillin resistant staphylococcal species simultaneously using *nuc* and *phoP* genes. Amplification of *mecA* gene in 159 isolates confirmed that all strains are methicillin resistant, except UOM160 (MSSA) and multi-drug resistant (MDR) in 159 isolates confirmed by 22 sets of β-lactam antibiotics. MSSA and MDR-MRSA were discriminated by PCR-SSCP using *nuc* gene for the first time. From the present studies, compared to conventional methods MALDI-Biotyper emerged as an effective, sensitive (>99%), robust (<2 min), and alternative tool for pathogen identification, and we developed a PCR-SSCP technique for rapid detection of MSSA and MRSA strains.

## Introduction

*Staphylococcus aureus* ^1^ is recognized as one of the most common pathogens responsible for food poisoning and causing various infections in animal and humans^2^. This facultative anaerobe is a natural flora in 20–30% of people, present within the anterior nares and on the skin. Infection occurs mostly by evasion of immune system in the host to cause pneumonia, pulmonary tuberculosis, endocarditis, sepsis, soft tissue infections, toxic shock illness, bone and joint infections, or urinary tract infections and even food poisoning^3, 4^. *S*. *aureus* expresses distinctive surface proteins that are critical for binding to host cells to act as virulence factors. These surface proteins ordinarily promote attachment to laminin and fibronectin. Most strains also express a clumping factor, coagulase protein reaction, which promotes attachment to blood clots. As soon as the bacterium gets adhered, it multiplies into a biofilm that makes it difficult to eradicate^5^.

First incidence of methicillin-resistant *S*. *aureus* (MRSA) was reported in late 1960s. The MSSA strain acquiring genetic element ‘Staphylococcal cassette chromosome’ called *mec* (*SCC mec*) which reduces affinity to the group of β-lactam antibiotics by encoding the altered penicillin-binding protein 2a -PBP2a expressed by *mecA* gene and allowed bacteriocin to assemble cell wall in presence of drugs^4, 6^? Recent reports state that, acquisition of resistance in Staphylococci is by transposons and/or plasmids mediated by conjugate transfer, which spreads resistance elements among Staphylococci species. They can also mobilize conjugate plasmids to re-combine and form new plasmids then acquire and transfer potential resistant determinants^7–9^.

Although MRSA infection emerged in hospitals (Hospital acquired, HA-MRSA) called nosocomial pathogen, then it spread to community (Community acquired, CA-MRSA) and to livestock (Livestock associated, LA-MRSA). The CA-MRSA strains are frequently associated with young and healthy individuals who lack any predisposing risk factors for MRSA infection^3, 10^. Outbreaks of LA-MRSA in people without having any live stock contact shows that, this strain of the pathogen may transmit through food chain^10^. MRSA is the potential risk factor for humans, transmitted via food handling/consumption^11^.

Identification of pathogens by conventional methods of biochemical assays and phenotype determination are time consuming, costly and may lack capability to distinguish between several *Staphylococcal* species^12^. 16 S rRNA and 18 S rRNA sequencing based on polymerase chain reaction (PCR) and micro-nuclear magnetic resonance (µNMR) sequencing are promising and highly sensitive for identification at species level, but they are costly and time consuming^12^. Recently matrix-assisted laser desorption ionization time-of-flight mass spectrometry (MALDI-TOF-MS) has emerged as a rapid and robust tool for microbial identification and diagnosis. In this proteomic approach, microbes are identified using either intact cells or cell extracts. The process is rapid, sensitive, and economical because the data generated in MALDI-TOF-MS was compared against inbuilt proprietary database of well characterized reference strains^12, 13^.

The genetic evaluation performs a foremost role in biomedical fields (*e*.*g*., oncology and genetic ailments) for diagnostic functions. PCR-coupled single strand conformation polymorphism (PCR-SSCP) evaluation has been validated for the identification of species and strains in the absence of distinguishing morphological characters. The SSCP relies on the precept that, the electrophoretic mobility of single-stranded DNA molecule in a non-denaturing gel depends on its size and structure^14, 15^. The PCR–SSCP is a novel method, so far has not been used for discriminating food-borne pathogens in detecting mutations. For the first time PCR-SSCP has been by us to detect new mutations in *S*. *aureus*. Its speed and simplicity for detection of these/equivalent mutations make it more useful in clinical diagnostic laboratories^16^.

So far, despite consumption of vegetarian and non-vegetarian foods, no report is available on the prevalence and characterization of livestock associated coagulase positive Staphylococci (CoPS) MRSA. The objective of the present study was to establish robust MALDI-Biotyper™ a bench top microflex LT™ for mass spectrometry analysis) for pathogen identification and also methicillin susceptible *Staphylococcus aureus* (MSSA) and multi drug resistance (MDR)-MRSA discrimination. This was established for the first time using molecular tool PCR-SSCP as a simple method for point of care (POC) use.

## Results

### Screening and isolation of *Staphylococcus aureus* from food samples

In the present study 500 food samples were collected from different regions of Mysuru area, Karnataka, India and screened for food-borne Staphylococci. Food samples collected from Devaraj Urs road and Bamboo bazaar areas are highly contaminated as compared to others. The main source of contamination is possibly the population crowd of the area arriving from different environments (Supplementary Fig. S1, Tables S1–S6). The food-borne pathogens isolated from food include 160 ± 0.01 *Staphylococcal* spp., 92 ± 0.03 *Escherichia coli*, 108 ± 0.01 *Bacillus cereus*, 85 ± 0.11 *Salmonella typhimurium*, 29 ± 0.04 *Shigella flexneri*, 20 ± 0.04 *Vibrio cholera* and 8 ± 0.02 *V*. *parahaemolyticus* on selective Baired Parker Agar (BPA) media, Eosin methylene blue (EMB), Bacillus Cereus Agar (BCA), Xylose Lysine Deoxycholate (XLD), Deoxycholate Citrate Agar (DCA) and Thiosulfate-Citrate-Bile Salts (TCBS) respectively. Among 160 *Staphylococcal* spp., 152 isolates were confirmed as *S*. *aureus* (95 ± 0.02%), 6 as *S*. *epidermidis* (3.75 ± 0.01%) and 2 confirmed as *S*. *haemolyticus* (1.25 ± 0.03%) (Supplementary Tables S6 and S7, Fig. S2A). This shows, the occurrence of food contamination is high in processed, vegetarian, non-vegetarian foods, bakery, and dairy products was observed (Supplementary Fig. S1A). The total 500 samples were screened and isolated 502 pathogens on selective agar by repeated thrice for concordant confirmation, and significant results (*P* < 0.03) confirmed the role of selective media in isolating of food-borne pathogens.

### Identification of *Staphylococci* species

#### Conventional methods

All 160 *Staphylococcal* isolates were characterized as Gram-positive bacteria; further biochemical test results are provided in supplementary Table 2. Out of 160 isolates, 152 were coagulase positive *S*. *aureus* (95 ± 0.02%, *P* < 0.001), 6 were recorded as coagulase negative- *S*. *epidermidis* (3.75 ± 0.01%, *P* < 0.001), 2 were *S*. *haemolyticus* (1.25 ± 0.05%, *P* < 0.01), (Supplementary Fig. S2) and these are statistically significant when compared to conventional sequencing. The extracted bacterial DNA samples were used for amplification of 16 S rRNA by PCR, amplicons were sequenced, submitted to GenBank and the accession numbers obtained were (*S*. *aureus-*KX355577, *S*. *epidermidis-*KX371861, *S*. *haemolyticus-*KX371862, *E*. *coli-*KX452114, *B*. *cereus-*KX641607, *S*. *typhimurium*-KX452116, *S*. *flexneri-*KX452115, *V*. *cholera-*KX452112, *V*. *parahaemolyticus-*KX452113 and *Enterobacter aerogenes-*KX452117 isolates). The conventional biochemical and sequencing results matches 50% of results (50% non reductant due to sample preparation and analysis conditions) between the methods and shows statistically significant (*P* < 0.05) while identifying the pathogens.

#### Biotyper: MALDI-TOF-MS method

Fourty-four *Staphylococci* were used for MALDI-Biotyper based on 8 MDR pattern. All the 44 common *Staphylococcal* spp., including MRSA, MSSA, methicillin resistant *Staphylococcus epidermidis* (MRSE), methicillin resistant *Staphylococcus haemolyticus* (MRSH) were identified by direct colony smear (DCS) and 70% formic acid protein extraction methods. Suspending 1–5 bacterial colonies for 70% formic acid extraction resulted in higher score (>2.0) for species level identification, but we could not recognize and retrieve species level quality of the spectrum by suspending high bacterial cell concentrations (3 × 10^10^ cells/mL). Compared to protein extraction method, DCS method is fast and retained significantly good results. The protein extracted for 44 samples were 90% correlated (*P* < 0.04 significant) to the DCS method (*P* < 0.02) having 100% matching to related species, this significantly (*P* < 0.001) compared to the biochemical and gene sequencing methods. The 10% relative error occurred was identified in protein sample preparation and due to higher bacterial load in DCS. This expresses MALDI-Biotyper method is more reliable compared to the conventional methods to treat targeted pathogens before they make adverse conditions.

#### MALDI-TOF-MS analysis

**Figure 1:**
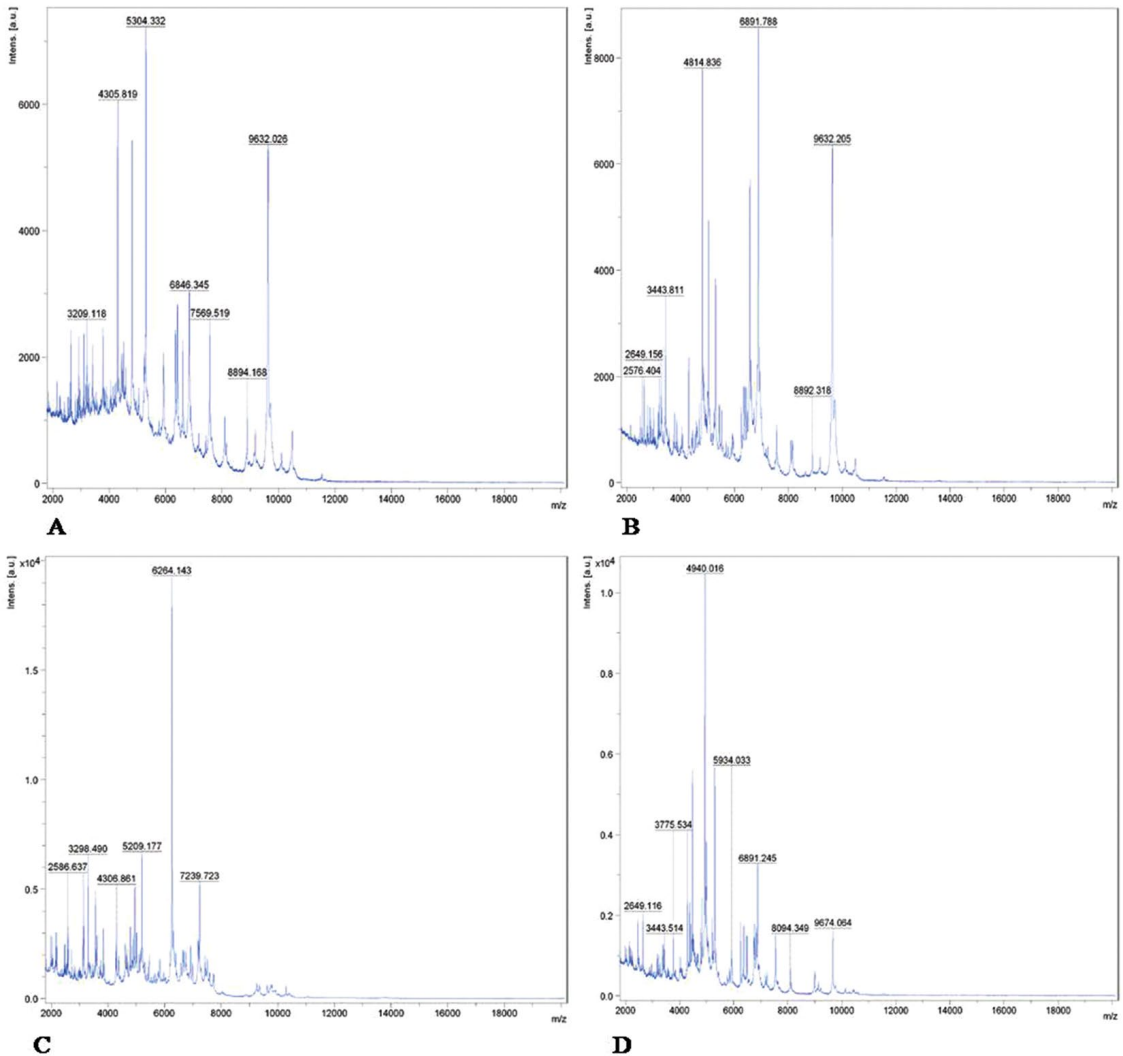
A typical MALDI-TOF-MS profile **(A–D)**. Representative protein spectra of (**A**). MSSA (UOM160), (**B**) MDR-MRSA (UOM090), (**C**) MRSE (UOM036) and (**D**) MRSH (UOM146) respectively. Total 44 *Staphylococcal* isolates used for identification by MALDI-Biotyper using DCS and protein extraction methods. Biotyper efficiently identified all 44 *Staphylococcal* isolates into methicillin resistant *S*. *aureus* (MRSA), *S*. *epidermidis* (MRSE) *and S*. *haemolyticus* (MRSH).

Among 58 isolates submitted for MALDI-TOF-MS analysis, 44 *Staphylococcal* spp., and 14 other food-borne pathogens were confirmed (Table 1). The higher score was obtained for UOM90 (2.28) followed by UOM150 (2.23) and UOM57 (2.20) isolates of *Staphylococcal* spp., by MALDI-Biotyper. It was possible to identify accurately all 58 (100%) isolates by comparision to Bruker inbuilt MALDI-Biotyper database. Fifty two isolates of *Staphylococcal* spp., had score value ≥2.0, and 7 other food-borne pathogens had 1.70–1.99 score (Table 1). MALDI-Biotyper accurately distinguished food-borne pathogens by proteins m/z ratio. The unique m/z ratio in *Staphylococcal* spp., identified was 4305.8 and also within the genus level identified, distinct m/z ratio was for MRSA-9632.2, MSSA-9725.8, MRSE-7239.7 and MRSH-9674.0 (Fig. 1A–D, Supplementary Fig. S3). The obtained same concordant analysis m/z ratio results confirms its repeatability of the experiment was significant (*P* < 0.001) to other identification methods compared with time taking for identification (within 2 min).

**Table 1:** Results of MALDI-Biotyper identification.

Reference ID	No.	Matched ID with score ≥2.00		Matched ID with score at 1.70–1.99		Total matched ID with score ≥1.70		Unmatched ID at 1.70–1.99		Unmatched ID with score ≥2.00		Total un matched	
		Number	%	Number	%	Number	%	Number	%	Number	%	Number	%
*S*. *aureus*	36	34	94.4	2	5.5	36	100						
*S*. *epidermidis*	6	5	83.3	1	16.6	6	100						
*S*. *haemolyticus*	2	2	100	—	50	2	100						
*S*. *typhimurium*	2	2	100	—	50	2	100						
*S*. *flexneri*	2	2	100	—		2	100						
*E*. *coli*	2	2	100	—		2	100						
*B*. *cereus*	2	2	100	—		2	100						
*P*. *aeruginosa*	2	2	100	—		2	100						
*V*. *cholera*	2	1	50	1		2	100						
*V*. *parahaemolyticus*	1	1	50	1		2	100						
*E*. *aerogenes*	1	1	100	—		1	100						
Total all species	**59**	**52**	**88.1**	**7**	**13.4**	**59**	**100**	**0**		**0**		**0**	

Total 59 food-borne pathogens used for MALDI-Biotyper identification and results were compared to MALDI-Biotyper data base then matched reliable ID used for genus and species level identifications.

#### Identification by conventional methods versus MALDI-TOF-MS method

Forty four isolates used for MALDI-Biotyper belonged to *Staphylococcal* spp., which included *S*. *aureus*, *S*. *epidermidis* and *S*. *haemolyticus* (Table 1). Results are reported as numeric score based on collective comparison of protein spectra obtained experimentally compared to MALDI Bruker’s Biotyper-specific database. Scores below 1.69 reported as non-reliable genus ID, scores of 1.70–1.99 were classified as probable genus ID, scores of 2.00–2.29 were secure genus ID and scores of 2.30–3.00 designated as highly probable species ID. Compared to conventional methods, MALDI-Biotyper confirmed higher scores for *Staphylococcal* isolates within 2 min like similar to 16 S rRNA sequencing results. This indicated the advantage including les time consuming, economical, and sensitivity wise MALDI-Biotyper method is >99% more sensitive (*P* < 0.001), distinguishable in comparison to conventional methods for point of applications. In conventional biochemical methods judging at species level is highly difficult, no accuracy and no significance between the results due to change in the experimental conditions. But, sequencing is accurate compared to biochemical (100%, *P* < 0.05) and MALDI-biotyper is100% sensitive (*P* < 0.001) compared to other any methods.

#### Ambiguous and erroneous identification by MALDI-TOF-MS

One isolate was misidentified by biochemical tests. MALDI-TOF-MS assuming at least a high score of 2.00 is necessary for accurate identification of food-borne pathogens. Among them n = 44 isolates, the score value obtained more than 2.00 (86.36 ± 0.03%, n = 38/44) and 2.20 (13.64 ± 0.0%, n = 6/44) revealed the identification at species level without any ambiguity. Culturing and by repeated biochemical tests identified wrongly the *Enterobacter aerogenes* as compared to MALDI-TOF-MS. In biochemical method, sometimes Indole and Methyl red tests are likely to give false positive results due to slight alteration in pH at analysis time with respect to *E*. *coli*. This data suggest that, MALDI-Biotyper is 100% (*P* < 0.001) accurate compared to conventional methods by identifying 58 analyzed including *E*. *aerogenes* both in protein extraction and DSC methods in the present study.

### Detection of *Staphylococcal* species by *nuc* genes and mPCR

**Figure 2:**
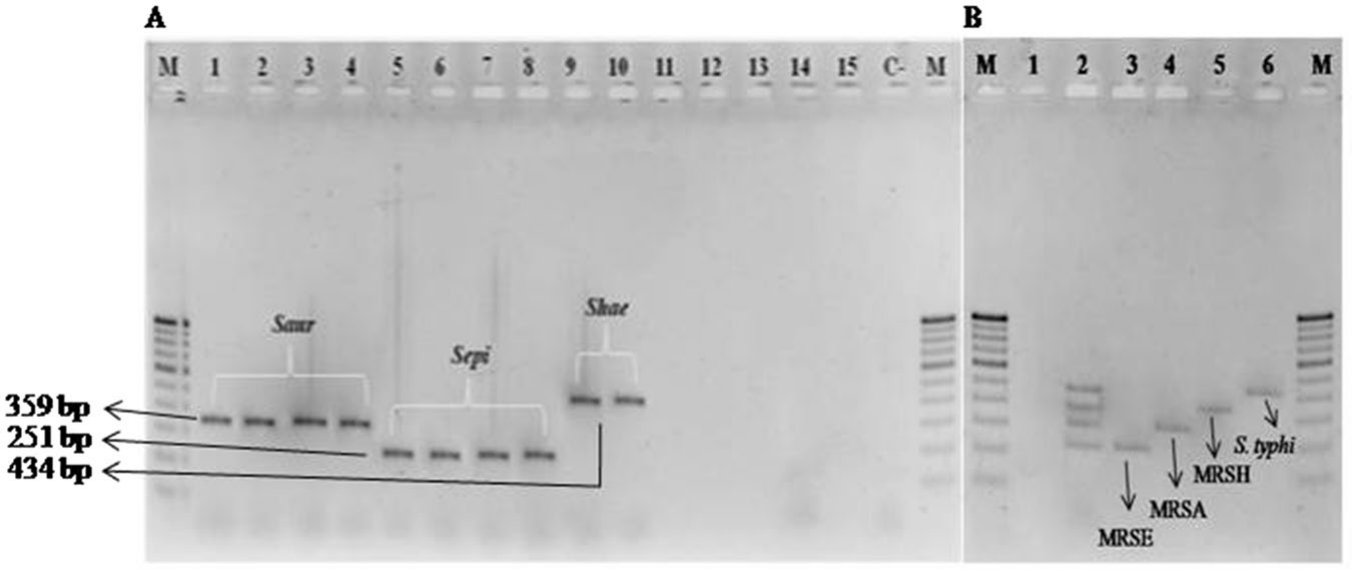
(**A**) MDR *Staphylococcal* strains discriminated by universally conserved thermonuclease (*nuc*) gene. Isolated 160 Staphylococcal spp., were screened to identify at species level by PCR using universally conserved thermonuclease (*nuc*) gene. PCR confirmed as 151 methicillin resistant *S*. *aureus* (Lane 1–4, 359 bp), 6 as *S*. *epidermidis* (Lane 5–8, 251 bp) and 2 as *S*. *haemolyticus* (Lane 9 and 10, 434 bp) used along with *E*. *coli* (Lane 11), *S*. *typhimurium* (Lane 12), *S*. *flexneri* (Lane 13), *V*. *cholera* (Lane 14), and *V*. *parahaemolyticus* (Lane 15) were compared with 100 bp ladder (M) and water as negative control (C-). (**B)** Illustration of PCR amplicons expected when using the updated *Staphylococcal* spp. multiplex PCR. Lane M, 100 bp ladder; Lane 1, negative control (C-); Lane 2, mixture of multiplex PCR products obtained for all the templates of MDR-MRSA, MRSE, MRSH and *S*. *typhimurium;* Lane 3, MRSA; Lane 4, MRSE*;* Lane 5, MRSH and Lane 6, *S*. *typhimurium* comparable to the amplified product of mPCR in lane 2.

All the 160 *Staphylococci* isolates were subjected to PCR using universally conserved thermonuclease (*nuc*) gene present in all *Staphylococcal* species except *S*. *sciuri*. Specifically designed *Saur*, *Sepi* and *Shae* (Table 2) primers were used for discrimination of *S*. *aureus*, *S*. *epidermidis* and *S*. *haemolyticus* isolates, respectively. The PCR successfully amplified the DNA fragments corresponding in size to each species as follows: *S*. *aureus*, 359 bp; *S*. *epidermidis*, 251 bp; and *S*. *haemolyticus*, 434 bp (Fig. 2A) as compared to *E*. *coli*, *S*. *typhimurium*, *S*. *flexneri*, *V*. *cholera*, *V*. *parahaemolyticus* pathogens, in the experiment. Simultaneously also detected *S*. *aureus*, *S*. *epidermidis*, and *S*. *haemolyticus* food-borne pathogens by mPCR using *Saur*, *Sepi*, *Shae* and *phoP* primers compared to water as negative control (Fig. 2B). Apart from nucleic acid extraction, the PCR and mPCR efficiency depends on primer designing for target gene detection and here it show 100% significant (*P* < 0.001) results in any PCR based detections by detecting 160 *Staphylococcal* isolates. Compared to other methods, known specific target detections validate the assay to be the best based on concordant results.

**Table 2:** Different primers used in the present study.

Sl. No.	Gene/region Name	Primer designation	5′→3′	Annealing temperature (°C)	Molecular weight in bp	Reference
1	*16 S rRNA*	*16 S rRNA* F	TGGTAGTCCACGCCCTAAAC	56	210	[55]
		*16 S rRNA* R	CTGGAAAGTTCCGTGGATGT			
2	*nuc*	*Saur* F	TCGCTTGCTATGATTGTGG	58	359	[20]
		*Saur* R	GCCAATGTTCTACCATAGC			
3		*Sepi* F	TTGTAAACCATTCTGGACCG	58	251	
		*Sepi* R	ATGCGTGAGATACTTCTTCG			
4		*Shae* F	TAGTGGTAGGCGTATTAGCC	58	434	
		*Shae* R	ACGATATTTGCCATTCGGTG			
5	*Eap*	*Eap* F	TACTAACGAAGCATCTGCC	62	230	[60]
		*Eap* R	TTAAATCGATATCACTAATACCTC			
6	*Coa*	*Coa* F	AAGATGGCACAGTATCATATGG	58	230	This study
		*Coa* R	GCCATATGTCGCAGTACC			
7	*SpA*	*SpA* F	AGCACCAAAAGAGGAAGACAAC	58	304	This study
		*SpA* R	ATGTACTCCGTTGCCGTCTT			
8	*mecA*	*mecA* F	TCCAGATTACAACTTCACCAGG	53	162	[50]
		*mecA* R	CCACTTCATATCTTGTAACG			
9	*PVL*	*luk-PV-1* F	ATCATTAGGTAAAATGTCTGGACATGATCCA	58	433	[52]
		*luk-PV-2* R	GCATCAASTGTATTGGATAGCAAAAGC			
10	*Stx*	*stx F*	ACACTGGATGATCTCAGTGG	57	614	[57]
		*stx R*	CTGAATCCCCCTCCATTATG			
11	*ompW*	*ompW F*	CACCAAGAAGGTGACTTTATTGTG	59	588	[56]
		*ompW R*	GAACTTATAACCACCCGCG			
12	Scaffolding protein	*Sf F*	TCTCCGCGACAGAAATCACT	56	204	This study
		*Sf R*	CCTGAACTGGACCCACTCAT			
13	phoP	*phoP F*	ATGCAAAGCCCGACCATGACG	56	299	[59]
		*PhoP R*	GTATCGACCACCACGATGGTT			
14	Emetic toxin	*Bc F*	GACAAGAGAAATTTCTACGAGCAAGTACAAT	58	635	[61]
		*Bc R*	GCAGCCTTCCAATTACTCCTTCTGCCACAGT			
15	*toxR*	*toxR F*	GTCTTCTGACGCAATCGTTG	56	368	[58]
		*toxR R*	ATACGAGTGGTTGCTGTCATG

### Confirmation of phenotypic virulence factors

In the present study, the 160 *Staphylococcal* species showed γ-hemolytic and α -hemolytic properties. The 121 *S*. *aureus* (75.62 ± 0.04) showed α-hemolytic property and 3 *S*. *epidermidis* (1.87 ± 0.02) and 2 *S*. *haemolyticus* (1.25 ± 0.02) expressed the γ-hemolytic activity. The present investigation highlights that the isolated food-borne pathogens can harm in Mysuru region of Karnataka, India. The evaluated 160 pathogens confirm the phenotypic virulence (100%) in isolates based on reaction observed on blood agar medium and repeated experiments significantly (*P* < 0.02) correlates the method tested in the present study.

### MRSA/MSSA

Out of 500 food samples, 159/160 (94.37 ± 0.02%) were positive for MRSA which is confirmed by targeting *mecA* gene responsible for resistance in *Staphylococci* species located on mobile genetic element called *SCCmec*, except in UOM169 (MSSA) isolate (Supplementary Fig. S4). In the present study none of the 152 MRSA (95 ± 0.04) isolates carried genes for production of Panton-Valentine Leukocidin (PVL) which indicates that, isolates enumerated strictly from food and not from HA- and CA- associated with diseased human or previously exposed to hospital treatment. The PCR analyzed for *mecA* gene targeting resistant gene express its efficacy by discriminating the resistant and susceptible isolates significantly (*P* < 0.001) and judged specific identification is 100% which is not able to distinguish by conventional and biotyper methods. In this way, the *mecA* gene targeted identification confirms and stand best compared to culture, biochemical, and Biotyper method.

### Species specific detection of MRSA

Totally 152 MRSA isolates were identified by specific MRSA *Coa*, *SpA*, *Eap* genes. The amplified DNA amplicons separated by electrophoresis and product size corresponds to specific genes *Coa* (290 bp), *SpA* (304 bp) and *Eap* (230 bp) comparing to other species *E*. *coli*, *S*. *typhimurium*, and *S*. *flexneri* (Supplementary Fig. S5). Other species did not show any response to these genes; this confirmed MRSA and synthesized primers for unique determinants present in MRSA but not in other *Staphylococcal* spp. These specific primers (*Coa*, *SpA* and *Eap*) matches the 100% identification but compared to conventional methods, PCR is accurate in nature by identifying at species level (*P* < 0.01) in short period of time.

### Phenotypic antibiotic resistance and MDR profile

**Figure 3:**
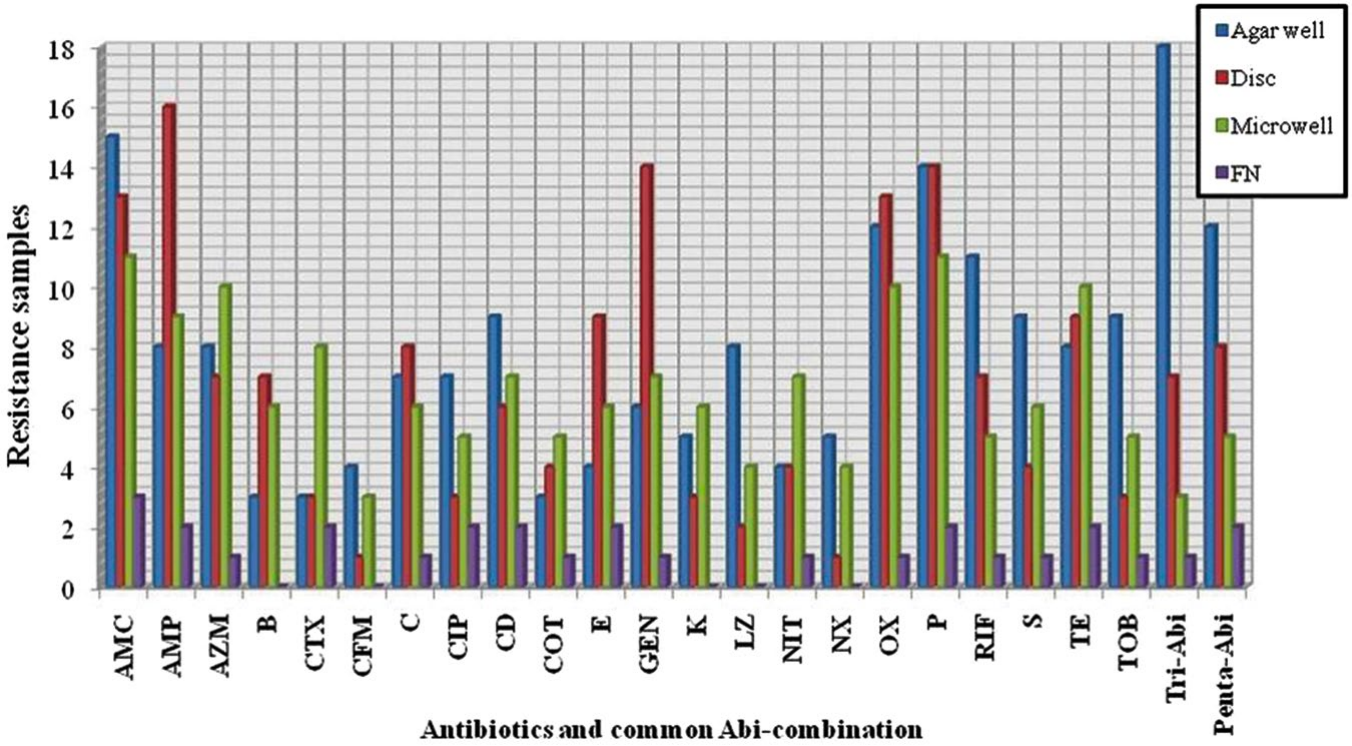
Susceptibility prediction test for *S*. *aureus*. Total 159 *Staphylococcal* spp., screened for antimicrobial susceptibility using agar well, disc diffusion and microwell methods. Proportions of MDR *S*. *aureus* samples correctly identified as resistant by agar well (blue), disc (red), microwell (green) compared with false negative (purple). Abbreviations: AMP- Amphicilin, AZM-Azithromycin, AMC- Amoxyclav, B- Bacitracin, CFM- Cefoxitin, CTX- Cefotaxime, C- Chloramphenicol, CIP- Ciprofloxacin, CD- Clindamycin, COT- co-Trimoxazole, E- Erythromycin, GEN- Gentamycin, K- Kanamycin, LZ- Linezolid, NIT- Nitrofurantoin, NX- Norfloxin, OX-Oxacillin, P- Pennicilin, RIF- Rifampicin, S- Streptomycin, TE- Tetracycline, and TOB- Tobramycin.

Among the 159 isolates, 14 classes of methicillin resistant *Staphylococcal* isolates were identified and they showed resistance to 22 antibiotics patterns significantly (*P* < 0.02) (Table 3, Supplementary Table S12). Results of antibiotic susceptibility carried out for 22 antibiotics by agar well, disc and micro dilution methods and they were presented in Fig. 3. Most of the isolates showed resistance to oxacillin (n = 137/159, 86.16 ± 0.02%, *P* < 0.03) and oxacillin + penicillin (n = 22/159, 13.83 ± 0.04%, *P* < 0.02) in which *mecA* gene (159 isolates-97.37 ± 0.01%, *P* < 0.01) harbored. To all the three methods minimum inhibitory concentration criteria was applied to categorize as resistant or susceptible as per the CLSI guidelines adopted in this study. To elucidate the molecular mechanism of resistance, each *Staphylococcal* specific gene was sequenced (KX371863, KX387571, KX387572). Representative MRSA (n = 97, *P* < 0.01) was resistant to amphicilin (AMC), azithromycin (AZM), amoxyclav (AMC); MRSE (n = 4, *P* < 0.001) was resistant to tetracycline (TE) and MRSH (n = 2, *P* < 0.03) was resistant to azithromycin (AZM), tetracycline (TE), kanamycin (K), and tobramycin (TOB). MRSA also showed mild resistance to other antibiotics. In the present investigations, the isolates MRSH > MRSA > MRSE showed decreasing order of resistant significantly (*P* < 0.02) according to compared evaluation of 22 antibiotics. This shows highly pathogenic nature of food-borne pathogens having determents for multiple antibiotic residents.

**Table 3:** Distribution of antibiotic resistance patterns among MDR-CoPS^a^ and CoNS^b^.

MDR strains		MDR groups	Class and number
*S*. *aureus* ^a^	UOM082	*S*, *TE*, *NIT*, *AMP*, *K*, *TOB*, *OX*, *P*, *CIP*, *C*, *AMC*, *AZM*, *B*, *CFM*	Class-14 (n = 95)
	UOM090	*CD*, *NIT*, *K*, *TE*, *AZM*, *B*, *NX*, *CTX*, *P*, *OX*, *RIF*, *S*, *C*, *TOB*	Class-10 (n = 12)
	UOM114	*NIT*, *AMP*, *TOB*, *RIF*,*COT*, *OX*, *P*, *C*, *CFM*, *B*	Class-8 (n = 21)
	UOM048	*AZM*, *B*, *CFM*, *E*, *OX*, *P*, *RIF*, *S*	Class-7 (n = 11)
	UOM018	*LZ*, *CIP*, *K*, *OX*, *P*, *C*, *CFM*, *B*	Class-5 (n = 2)
	UOM136	*TOB*, *RIF*,*COT*, *AMP*, *B*, *K*, *OX*, *P*	Class-4 (n = 3)
	UOM012	*AMP*, *AMC*, *CTX*, *OX*, *P*, *COT*,*TE*	Class-3 (n = 7)
	UOM051	*TE*, *NIT*, *K*, *TE*, *AZM*	Class-2 (n = 1)
	UOM069	*TE*, *OX*, *P*, *AMC*	Class-14 (n = 2)
	UOM157	*TOB*, *CD*, *CFM*	Class-11 (n = 2)
	UOM160^c^	*E*, *AMC*	Class-8 (n = 1)
*S*. *epidermidis* ^b^	UOM057	*S*, *TE*, *NIT*, *K*, *AMP*, *TOB*, *OX*,*COT*, *CIP*, *C*, *AMC*, *AZM*, *B*, *CFM*	Class-7 (n = 1)
	UOM036	*S*, *TE*, *B*, *CFM*, *OX*, *K*, *P*, *RIF*, *S*, *AMC*, *CTX*	Class-12 (n = 1)
	UOM080	*NIT*, *AMP*, *TOB*, *CFM*, *E*, *OX*, *S*, *TE*	Class-10 (n = 1)
	UOM097	*TE*, *AZM*, *RIF*,*COT*, *AMP*, *B*, *CFM*	
*S*. *haemolyticus* ^b^	UOM142	*NIT*, *TE*, *AZM*, *K*, *B*, *CFM*, *CTX*, *AMP*, *TOB*, *CFM*, *E*, *OX*	
	UOM150	*TOB*, *COT*, *C*, *K*, *NIT*, *TE*, *AZM*, *E*, *AMC*, *OX*, *P*	

Isolated 160 food-borne pathogens screened for antibiotics test using 22 different antibiotics. Antibiotic resistance pattern showed by *Staphylococcal* isolates were grouped and results represented by one individual strain from the MDR group. MDR (multidrug-resistance) coagulase positive^**a**^, coagulase negative^**b**^ *Staphylococcal* resistance to antibiotics belonging to three or more class; n-number of isolates and^**c**^ *mecA* gene negative isolate. Abbreviations: AMP- amphicilin, AZM-azithromycin, AMC- amoxyclav, B- bacitracin, CFM- cefoxitin, CTX- cefotaxime, C- chloramphenicol, CIP- ciprofloxacin, CD- clindamycin, COT- co-Trimoxazole, E- erythromycin, GEN- gentamycin, K- kanamycin, LZ- linezolid, NIT- nitrofurantoin, NX- norfloxin, OX-oxacillin, P- pennicilin, RIF- rifampicin, S- streptomycin, TE- tetracycline, and TOB- tobramycin. The most frequently occurring phenotype in MDR stains was OX-P-TE (Tri-Abi) and AMP-AZM-AMC-RIF-TE (Penta-Abi).

Among the 151 *S*. *aureus* isolates, 148 (98.01 ± 0.03%) revealed multi-drug resistance (MDR) exhibiting resistance to synthetic antibiotics belonging to more than three classes. Among isolates resistant to more than one antibiotic majority of them were resistant to oxacillin (87.64 ± 0.02%) and penicillin (80.10 ± 0.01%). In the present study 16 representatives MDR isolates revealed simultaneous resistance to β-lactam antibiotics of second, third and fourth generation group of antibiotics. Besides synthetic β-lactam, all the 16 group MDR isolates have varying antimicrobial resistance pattern (Table 3) and also exhibited following special antimicrobial resistant profile: OX-P-TE (Tri-Abi) and AMP-AZM-AMC-RIF-TE (Penta-Abi) (Fig. 3). This Penta-Abi antibiotic pattern was influenced by presence of multiple determents controlling over there for such resistance. The evaluated 148 isolates and 16 special MDR isolates signifies (*P* < 0.02) the method 100% adaptive for screening a large number of isolates to understand the sensitiveness of pathogen and its effect.

### Easy discrimination of *S*. *aureus* by PCR-SSCP

**Figure 4:**
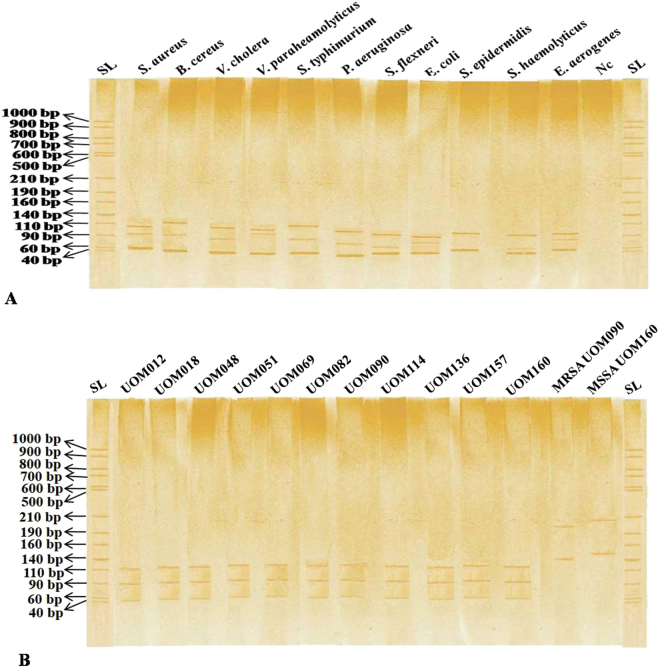
PCR-SSCP patterns of food-borne pathogens. The DNA was extracted, PCR-SSCP was performed and its finger prints were visualized on non-denaturing polyacrylamide gel. In Fig. A, different food-borne bacterial pathogens unique PCR-SSCP banding pattern and negative control compared to ssDNA ladder (SL). In Fig. 4B, PCR-SSCP finger prints of *S*. *aureus* (UOM 012, 018, 048, 051, 069, 082, 090, 114, 136, 157, and 160) isolates within the species compared. Again lane 13 and 14, LA-MRSA UOM090 and LA-MSSA UOM160 isolates containing thermonuclease gene was used to differentiate the resistant and susceptible pathogens and those special PCR-SSCP fingerprints were represented in Fig. B.

In the present study, MDR *Staphylococcal* isolates were analyzed at species level to discriminate resistance species by comparing other food-borne pathogens. Universally conserved 16 S rRNA region was used for discrimination of pathogens. The PCR amplified 16 S rRNA double stranded DNA of MDR-MRSA, MRSE and MRSH isolates were denatured and separated on non-denaturing polyacrylamide gel electrophoresis. PCR-SSCP finger prints were compared with other food-borne pathogens at genus level (Fig. 4A) and different species of *S*. *aureus* isolates were discriminated within the species (Fig. 4B). *Saur* primer sets for thermonuclease conserved gene in *S*. *aureus*, were clearly distinguished between MSSA and MDR-MRSA (Table 2). It is for the first time that this novel *nuc* region is used to discriminate resistant (MDR-MRSA) and susceptible (MSSA) isolates efficiently (Fig. 4B). The 148 analyzed pathogens in PCR-SSCP method significantly (*P* < 0.04) matches the discrimination of pathogens at species level compared to other methods in the present investigation. The single MSSA sample was determined ten times repeatedly for its *nuc* region signature and confirms its best nature of PCR-SSCP by determining phenotypic nature at species level for the first time compared to any other methods including conventional and MALDI-Biotyper based.

### MALDI-Biotyper and PCR-SSCP

The PCR-SSCP is ideal for the discrimination of single point mutations within bacterial genome. Compared with PCR, RFLP, and AFLP techniques, it provides high accuracy, sensitivity and capability to detect even single nucleotide difference within a mixed population of same species as done in this study. The MALDI-Biotyper has identified up to species level but not at genetic level present between the species (Supplementary Table S11). Even, though MALDI-Biotyper is sensitive and robust in identification of bacteria at species level but PCR-SSCP could do molecular diagnostic of *Staphylococci* MRSA and MSSA at phenotypic level was highlighted able (Fig. 4B). In this study, *Saur* primer used, could determine at phenotypic level by using conserved thermonuclease gene efficiently (*P* < 0.01) which is otherwise not confirmed by MALDI-Biotyper (*P* < 0.001). The total 58 isolates of different bacterial pathogens, 44 isolates were confirmed as *Staphylococcal* sp. which is confirmed by MALDI-Biotyper (*P* < 0.001). Those isolates discriminated by PCR-SSCP, showing 99% (*P* < 0.001) match between the methods except 1% (*P* < 0.02) deviated due to condition and samples preparation at which experiment carried out. MALDI-Biotyper is sensitive and robust in identifying the pathogens both at genus and species level. Compared to conventional time consuming methods, MALDI-Biotyper is more reliable for all the laboratories having high efficacy with low cost and discrimination of pathogens at phenotypic level can be achieved significantly in the present study.

## Discussion

MRSA is one of the major human pathogens associated with serious diseases, ranging from minor to life-threatening infections^17^. Public are unaware about the serious diseases caused by MRSA and in recent years, the spectrum of transmission routes have greatly varied. In the present study, 500 food samples were screened and isolated 511 ± 0.02 food-borne pathogens including *S*. *aureus*, *S*. *epidermidis*, *S*. *haemolyticus*, *E*. *coli*, *B*. *cereus*, *S*. *typhimurium*, *S*. *flexneri*, *V*. *cholera* and *V*. *parahaemolyticus* on specific media (Supplementary Fig. S1, Tables S1–S6). This study showed maximum (94.37 ± 0.02%) prevalence of MRSA in Mysuru region, Karnataka, India compared to other species (Supplementary Table S6). Doyle *et al*.^18^ highlighted vehicles involved in 573 *Staphylococcal* food poisoning outbreaks during 1998–2010; These included raw meat, sea food, vegetables and prepared dairy products. Present study also revealed that vegetarian, non-vegetarian, dairy products and ready-to-eat foods are the main transmission routes for Staphylococcal spp., specifically *S*. *aureus*. This indicates that improper food handling practices act as potential food vehicle for MRSA transmission and cause harmful effects on consumers^19^ and common practices, equipment, processing, and handling could lead to MRSA contamination at multiple levels in food chain.

The specific identification of MRSA at species level is very important because of two reasons, first is the public health associated with MRSA and secondly, acquiring antibiotic resistance in *Staphylococci* spp., differ from species to species which may mislead in providing proper treatment^17, 20^. According to Chajęcka *et al*.^9^ food samples contaminated with coagulase negative *Staphylococcal* species during handling or production process are the main source of the disease (Supplementary Table S8). Apart from CoNS, CoPS in the present study showed highest association with food in Mysuru. This indicates that, contamination by different routes is not properly known as also different types of food and these may be one the primary reasons for cross contamination or food spoilage.

Detections rely on traditional bacterial culture procedures that employ serial enrichments in isolation on selective‐differential agar plates and the process takes up to 5 days to get a presumptive positive isolate. Confirmation relies on traditional biochemical testing of sugar and nutrient utilization media, which can take several days to complete. The 1 cfu/g sensitivity is needed for food samples in 24 h to adopt culture based methods for POC-system. Much work has been focused on POC technologies that are affordable, robust, easy to use, portable, and provide sufficient quantitative accuracy to enable clinical decision making. Isolated pathogens are confirmed by 16 S rRNA sequence, which are deposited in NCBI GenBank and accession numbers are obtained (Supplementary Table S8). Currently culture based method applied in basic research is difficult to adopt in POC-systems due to lack of sensitivity and robustness. Different methods involved in pathogen identification and discrimination were compared based on cost, robustness and sensitivity, and these are presented in Supplementary Table S10. To overcome these drawbacks, present study adapted MALDI-Biotyper system as an alternative identification method because of simple sample preparation for MALDI target plate by DCS and protein extraction using 70% formic acid methods^12^. MALDI-Biotyper identified all submitted 44 *Staphylococcal* species as compared to other food-borne pathogens (Table 1). In the present study 44 isolates were identified with Biotyper score of ≥2.28 ± 0.01 for MRSA, ≥2.10 ± 0.01 for MRSE, ≥2.01 ± 0.04 for MRSH isolates, which showed concordant values with biochemical and 16 S rRNA sequencing results (Supplementary Table S11). Study showed that, DCS method is faster and simple for identification as compared to conventional method but there is inconsistency in protein extraction method. This evidence is, particularly for Gram-positive organism as the cell wall is not efficiently disrupted and it requires more time than DCS method. One study by Bizzini *et al*.^21^ showed that out of 1,373 isolates, 75% were identified by DCS and remaining 25% were further identified by ethanol/70% formic acid protein extraction method. This evidence clearly supports the present study on MALDI plate by DCS for identification than the protein extraction method. The biotyper system score is one of the important factors for identification of an isolate. Higher score indicates greater accuracy of identification. Present study showed that the use of 1 × 10^3^ cfu/mL bacterial cell suspensions for MALDI-Biotyper is recommended to achieve better results.

**Figure 5:**
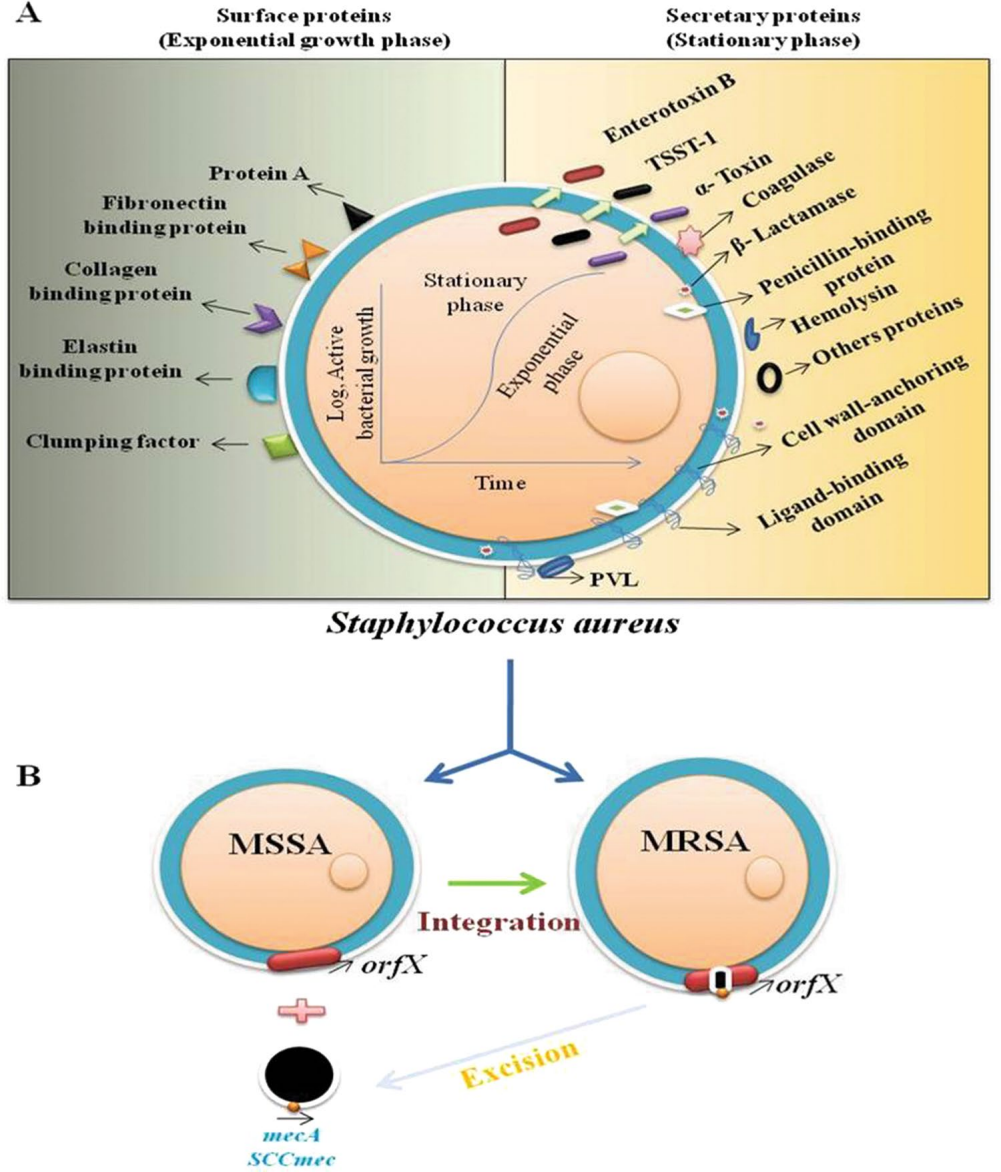
Proposed study showed *S*. *aureus* associated virulence factors and resistance mechanism. (**A**) Number of virulence factors present on surface, secreted in exponential and stationary phase during organism life span. 1. On surface different virulence factors called adherence determinant comprises of proteins covalently anchored to cell peptidoglycans are *Staphylococcoal* protein A (SpA), elastin-binding protein, fibronectin-binding protein A and B (FnbpA and FnbpB), collagen-binding protein and clamping factor (Clf) A and B proteins. 2. Many group of exoproteins such as (a). secretary toxins (toxic shock syndrome toxin-1, *Staphylococcal* enterotoxins (SEA to SE), exfoliative toxins A and B (ETA and ETB), pyrogenic toxin superantigens (PTSAgs), α/β/γ-hemolysin, leukocidin, Panton-Valentine leukocidin (*PVL*) proteins vigorously stimulate T-lymphocytes to proliferate, toxic shock syndrome and food poision outbreaks) (b) enzymes including proteases, lipases, collagenase, hyaluronidase, β–lactamase, nucleases and mainly coagulase involved in converting host tissue nutrients to use for its growth, and (c) other proteins also involved in tolerating host innate and adaptive immune system including staphylococcal complement inhibitor (SCIN), staphylokinase (SAK), chemotaxis inhibitory protein of *S*. *aureus* (CHIPS), extracellular fibrinogen binding protein (Efb), extracellular adherence protein (Eap) and formyl peptide receptor-like-1 inhibitory protein (FLIPr). These multifactorial virulence factors designed in *S*. *aureus* machinery system and express at different stages of infectious conditions to act efficiently. (**B**) Methicillin susceptible *S*. *aureus* develop resistance to number of synthetic antibiotics by acquiring resistance machinery *Staphylococcal* Cassette Chromosome *mec* (*SCCmec)* harboring gene called ‘*mecA’* integration to become methicillin resistant (called MRSA) and vice versa. This confirms and establish as a ‘central dogma’ to characterize strains to judge MRSA or MSSA.

PCR methods were attempted for specific identification and all *Staphylococcal* species were characterized for methicillin resistance gene *mecA* and other specific genes (Table 2, Supplementary Figs S4 and S5) such as *Coa*, *SpA*, *Eap* ^22^. Among 160 *Staphylococcal* isolates 159 (99.37 ± 0.04%) were confirmed as methicillin resistant and 152/160 were *S*. *aureus*. The *nuc* primers (Table 2) in mPCR were simultaneously confirmed as MRSA, MRSE, and MRSH. Out of 160 isolates 151 (94.34 ± 0.02%) isolates are MRSA and 6 (3.75 ± 0.01%) MRSE, and 2 (1.25 ± 0.05%) MRSH (Fig. 2B). Our results are supported by the results of Hirotaki *et al*.^23^, Who carried out mPCR to detect human-associated 361 *Staphylococcal* strains using *nuc* genes. Several virulence determinants such as lipases, hemolysins, thermonuclease, and hyaluronidase were implicated in pathogenesis of *Staphylococcal* spp., which involved in host cell invasion^24, 25^. PCR and MALDI-Biotyper in the study specifically detects the Staphylococcal isolates and showed significant correlation between the methods. In the present study, 160 *Staphylococcal* isolates (28.75 ± 0.03%) (n = 46) are virulent isolates and it is in accordance with the report of Pereira *et al*.^26^ and Cuvalova *et al*.^27^ who revealed common virulence factor associated with human infections involved in disruption of host cell membrane proteins lead to β-barrel like pores in cell membrane, which leads to leakage of cellular contents by cell damage. Apart from these virulence factors other cumulative virulence determinants also play important role in infections and food pathogen outbreaks (Fig. 5A; Supplementary Figs S6 and S7). Presence of these virulence determinants had a great impact on public health and clinical treatment. All 160 methicillin resistant *Staphylococcal* isolates showed negative results for PVL gene and confirmed that all *Staphylococcal* isolates were, isolated only from livestock but not from CA or HA (Supplementary Fig. S2). Epidemiological evidence shows that MRSA is increasing seriously infections in human’s beings^28^. PVL (β-pore forming exotoxins) is one of the important cytotoxins released by *S*. *aureus*, which creates pores mainly in host defense cell membranes of white blood cells (WBC), macrophages, and monocytes and causing severe leukocyte destruction, skin and tissue necrosis. This lethal toxin gene is typically associated with CA-MRSA strains but absent in LA-MRSA. This study significantly agrees with that of Battisti *et al*.^29^; Normanno *et al*.^10^ showed MRSA from food samples did not encounter PVL gene.

Limited information is available pertaining to diversity of *Staphylococcal* spp., which are resistant to antibiotic. In this context it is necessary to give attention to food associated CoPS and coagulase negative *Staphylococcus* (CoNS) strains which become resistant to more synthetic antibiotics. In the present study MR-SA, SE and SH screened for MDR pattern using 22 β-lactam antibiotics (Fig. 3) showed major resistance pattern for oxacillin-penicillin-tetracyclin ((OX-P-TE) called Tri-Abi) and amphicillin-azithromycin-amoxyclav-rifampicin-tetracyclin ((AMP-AZM-AMC-RIF-TE) called Penta-Abi) antibiotics (Table 3 and Fig. 3) among the studied isolates. The description of *S*. *aureus* isolated genes explored in Supplementary Table S12. These findings are in concordant with Chajecka *et al*.^9^ on antimicrobial MDR profile observed in MRSE and MRSH. Presence of MRSH in meat, cheese, fish, milk and wine indicates unhygienic condition present during processing^30, 31^. From the clinical point of view, methicillin-resistant CoPS and CoNS strains pose very serious therapeutic problem and it is due to acquired resistant *mecA* or *mecC* genes into MSSA (Fig. 5B) mobile genetic element called *SCCmec* cassette^4, 9^, which inturn enable to accumulate cell wall components of bacteria in the presence of antibiotics^32, 33^. Resistance to β-lactam antibiotics in *S*. *aureus* mediated by target site replacement by *mecA* or *mecC* genes encode substitute penicillin binding protein (PBP) 2a and for enzymatic inactivation of *blaZ*-encoded β-lactamase. Presently MDR was prevalent in isolates, from vegetarian, non-vegetarian, dairy products, and ready-to-eat foods which displayed resistance to two or more classes of antimicrobials. MRSA emerged as resistant to majority of semi-synthetic β-lactam antibiotics (methicillin)^34^. These resistant microbes protect from many classes of antibiotics and this was strongly matching with antibiotic test results (Supplementary Table S8). Antimicrobials act as target agents and trigger microbial flora into varying genetic makeup which leads to become antibiotic resistant. This helps *Staphylococcal* spp., live on skin and mucosal environment, also there is more chance for susceptible *Staphylococcal* species to acquire resistance genes through mutation or horizontal gene transfer to survive in the presence of antibiotics^35, 36^. This study suggests that MRSA incidence and types differ according to geographical area, food production and handling. Compared to heterogeneity within *Staphylococcal* spp.; MRSA distribution in foods significantly showed acquiring resistance (route of resistance was not well known) could be one of the reason for microbial contamination high in Mysuru.

PCR-SSCP continued to be one of the best low cost and less time consuming methods for mutation detection in human diseases causing organisms^37^, viruses^38^, plant pathogenic bacteria^39^ and fungi^40^. For the first time the present study used PCR-SSCP technique for discrimination of food-borne pathogens MSSA, MDR-MRSA, MRSE and MRSH. The mechanism behind the SSCP involves, denatured product ssDNA change into three dimensional conformation inturn it affects the electrophoresis mobility not only due to length, but also molecular weight, formation of loops and folding. In the present study, universally conserved 16 S rRNA gene discriminated food-borne pathogens and documented by unique banding patterns. PCR-SSCP banding pattern showed unique for MRSA. Also, designed *Saur* primer for conserved thermonuclease gene in *Staphylococcal* spp., discriminated MRSA and MSSA. The results showed there is a unique pattern for resistant and susceptible *S*. *aureus* pathogens. The PCR-SSCP technique is very sensitive in separation of ssDNA and it is inversely proportional to the size of fragment (*e*.*g*., single base pair differences resolved 99% of the time for 100–300 bp fragments, >80% for 400 bp)^41^ at least as large 775 bp analyzed successfully^42^. Technical information regarding SSCP is insufficiently reported in literature. The report of Chandrashekar *et al*.^39^, mentioned after formamide denaturation 210 bp products resolved under 8% acrylamide gel between 234 to 603 bp. In the present study, we successfully discriminated food-borne pathogens at genus level (Fig. 4A), *S*. *aureus* at species level (Fig. 4B) by 210 bp product successfully and it is different unique pattern compared to existing reports, and MRSA (UOM090) and MSSA (UOM160) isolates compared using conserved thermonuclease gene (Fig. 4B).

Similarly, molecular methods such as 16 S rDNA sequencing and PCR-based detection may be more powerful for identifying pathogens but these remain time consuming and costly. The MALDI-Biotyper highlighted as alternative robust and sensitive tool for identification of *Staphylococcal* spp., efficiently at species level. Factors contribute to the Biotyper score such as cell wall rigidity, growth phase, and culture conditions including selective media, which may affect protein expression. The concentration of cells spotted also influence the efficacy of MALDI-TOF-MS identification. The larger undisrupted cell masses give rise to spectral background noise resulting in no score or species ID. Even though this method robust, it needs sufficient data to compare and identify correctly. The PCR-SSCP method is simple and cost effective; it can be used as molecular diagnostic tool. The unique banding pattern obtained at genus, species and phenotypic level (Fig. 4A,B) identified as highly sensitive and economical method compared to MALDI-Biotyper and other PCR based methods.

**Figure 6:**
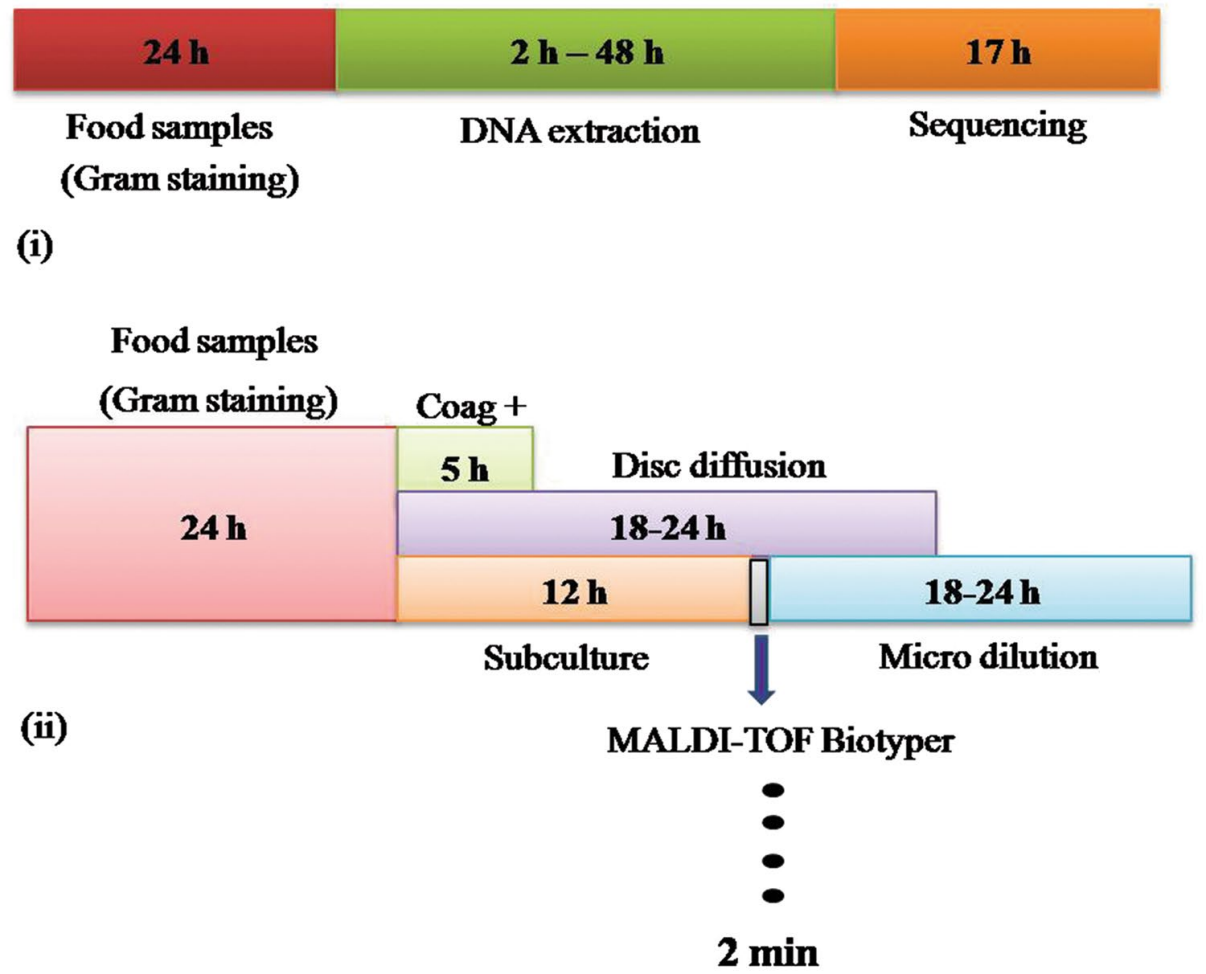
Schematic representation of work-flow of pathogen identification. The conventional method methods are time consuming and less sensitive for point of care applications. In the figure, (**i**) represents pathogen identification needs much time to identify the target pathogens, but the MALDI-Biotyper utilize only 2 min to identify the pathogen associated with food samples after enriching step was clearly presented (**ii**) and compared to other methods.

In the present investigation MALDI-Biotyper emerged as alternative, sensitive tool for pathogen identification and this stand to prescribe a target oriented proper treatment compared to other identification methods (Fig. 6). Isolated *Staphylococcal* spp., including MRSA, MRSE and MRSH confirmed for presence of methicillin resistance responsible *mecA* gene and used mPCR for discriminate three *Staphylococcal* spp., at once. The study highlighted the emergence of multidrug-resistant *S*. *aureus* with co-resistance to penicillin, oxacillin, tetracycline, amphicillin and linezolid antibiotics leads to decline drug effectiveness against MRSA infection. Also PCR-SSCP emerged as simple, effective tool for food-borne pathogen for the first time by discriminating susceptible (MSSA) and resistant (MRSA) *S*. *aureus*. In future, need to describe genetic basis for the remarkable ability of MRSA to acquire multi-antibiotic resistance and propose a novel paradigm for future chemotherapy against the multidrug-resistant pathogen is interesting. Study conclude that, the high prevalence of MRSA in different regions of Mysuru promoted us to investigate food samples and from the study need to suggest that improper handling cause major *Staphylococcus aureus* outbreaks. So, need improvement in food handling to avoid infections and its exacerbation was warranted.

## Materials and Methods

### Study design

*Staphylococci* were isolated from 500 different food samples comprising 137 vegetarian items, 69 non-vegetarian items, 87 dairy products, 72 bakery and 135 processed products collected from bakery, market, hospital canteen, and street venders located in different regions of Mysuru area, Karnataka State, India (Supplementary Tables S1–S6). The collected food samples were chilled and transported to the laboratory immediately and enumerated within 10 h for food-borne pathogens.

### Bacterial strains

All reference bacterial strains were received from Microbial Typing Culture Collection (MTCC), Chandigarh, India and American Type Culture Collection (ATCC) HiMedia, India. *Salmonella typhimurium* (98), *Escherichia coli* (1610), *Staphylococcus aureus* (96), *Bacillus cereus* (430), *Shigella flexneri* (1457), *Vibrio cholera* (3904), *Vibrio paraheamolyticus* (451), *Pseudomonas aeruginosa* (1688) and *Enterobacter aerogenes* (13048) strains were cultured as per the protocol prescribed by MTCC and ATCC. These strains were used as positive controls for identification of food-borne pathogens.

### Isolation, enumeration and identification of *S. aureus*

Food samples (25 g or 25 mL) were screened for *S*. *aureus* by transferring to 225 mL of buffered peptone water and mixed thoroughly^43^. The samples were incubated overnight at 37 °C for enrichment of cultures. Samples (0.1 mL) in Buffered Peptone Water (BPW) were streaked on to selective media plates containing Baired Prarker Agar^44^ and Mannitol Salt Phenol-red Agar^9^ and incubated overnight at 37 °C. BPA (+) and Mannitol (+) colonies were differentiated into coagulase positive and coagulase negative by detecting the production of coagulase on rabbit blood plasma and fibrinogen (RPF) medium^45^. Isolates were further confirmed as *S*. *aureus* by conducting biochemical tests like Gram staining, Kovac’s oxidase, KOH solubility, Coagulase, Catalase, Starch hydrolysis, Gelatin hydrolysis, Nitrate, Nipase and DNase tests as per the Manual of Clinical Microbiology^46^. Confirmed bacterial isolates were stored in 40% glycerol stock at −20 °C for further use.

### Extraction of genomic DNA

Overnight cultured bacterial isolates were centrifuged at 12,000 rpm for 10 min at 4 °C. The supernatant was discarded and pellet was subjected for DNA extraction using HiPurA^TM^ Bacterial Genomic DNA purification kit (Himeda, India) according to the manufacturer’s instructions.

### PCR and sequencing

The bacterial DNA were amplified by PCR using 16 S rRNA forword-*16S rRNA F* (TGGTAGTCCACGCCCTAAAC) and reverse-*16S rRNA R* (CTGGAAAGTTCCGTGGATGT) primers^39^ which are designed using primer 3 software. Amplification was performed in 0.2 ml PCR tube in volume of 25 µL containing 1 µL of 80–100 ng of genomic DNA, 1 µL of both forward and reverse primers (20 pmoL each) and 7 µL of DreamTaq Green PCR master mix (containing 0.25 mM each dNTP, 2 mM MgCl_2_ and Taq DNA polymerase) procured from Thermo Fischer Scientific, India. The PCR was performed in a master gradient thermal cycler (LABNET, NJ, USA) under following conditions: initial denaturation at 95 °C for 5 min; 30 cycles of denaturation for 30 sec at 94 °C, annealing for 30 sec at 56.6 °C, extension for 45 sec at 72 °C and final extension for 2 min at 72 °C followed by cooling to 4 °C until the sample was recovered. Amplified PCR products were confirmed on 1% agarose gel and image was captured in gel documentation system (BioRad, India). Amplified bacterial DNA products were sequenced at Eurofins Genomics, Bangalore, India and pathogens were confirmed by Basic Local Alignment Searching Tool (BLAST (http://blast.ncbi.nlm.nih.gov/Blast.cgi)).

### MALDI-TOF mass spectrometry

#### Sample preparation

The MALDI-TOF-MS analysis was done using a microflex LT™, bench top mass spectrometer (Bruker Daltonics, Bremen, Germany) in the Microbiological Laboratory, Tamil Nadu for identification of *Staphylococcal* species. Bacterial colonies were smeared onto stainless steel MALDI MSP target plate with and without application of 1 µL of 70% formic acid before adding 2 µL of Biotyper matrix solution. Bacterial protein was extracted by suspending varying concentrations of bacterial cells (10^3^ to 10^8^) into 300 µL deionized water in Eppendorf tube. Mixed thoroughly by adding 900 µL of ethanol and centrifuged at 13,000 rpm for 2 min, and dried the pellet at room temperature for 2–3 min. Further 1 to 5 µL of 70% formic acid and equal volume of acetonitrile (ACN) were added, vortexed and then sample was pelleted by centrifuging at 10,000 rpm for 2 min. Finally 1 µL of supernatant was added onto MALDI target plate and air dried at room temperature. Each spot was overlaid with 1 µL of HCCA solution, allowed to dry under fume hood at room temperature for 10 min. For each formic acid and direct sample to be analyzed was spotted in duplicate and spectra were recorded in positive linear mode within a mass range of 2–18 kDa.

#### Internal calibration and MALDI Biotyper sample analysis

The MALDI-Biotyper instrument (microflex) was first externally calibrated by dissolving α-cyano-4-hydroxy-cinnamic acid (HCCA, final concentration of 10 mg/mL) matrix substance (Bruker Daltonics, India) in standard solvent mixture comprising of acetonitrile 50%, trifluoroacetic acid 2.5% and water 47.5% (Sigma-Aldrich, India). As internal standard 10 pg/µL in 50% aqueous acetonitrile containing *Escherichia coli* ribosomal protein (Bruker Daltonics, India) was added to HCCA-matrix solution mixture and spotted on the designated calibration spots on the 384-well target plate. The MALDI-TOF-MS microflex LT™, bench top mass spectrometer (Bruker Daltonics, Bremen, Germany) was used in the linear mode. The mass spectrometer uses a 200-Hz frequency tripled Nd:YAG laser, operating at 355 nm. Ions generated by the MALDI process were accelerated at 20 kV through a grid at 19.3 kV into a short, linear, field-free drift region into the detector. For each 50 randomized spot, 40 sub-spectra (2000 spectra/spot) were collected and presented as one main spectrum. MALDI-TOF mass spectra were generated in the mass range of 2–20 kDa. Laser intensity was set between 3600 and 3800 V, obtaining signal intensities between 5 × 10^2^ and 1 × 10^4^ and internally compared to the inbuilt Bruker Biotyper version 3.0 database (with 5600 main spectrum profile (MSP)). This special precision makes MALDI-TOF-MS by determining differences in biomarker masses up to 1 Da according to manufacturer’s instructions.

#### Data processing: Evaluation of *S. aureus* by MALDI-TOF-MS Biotyper system

For automated data analysis, raw spectra data were processed using MALDI BioTyper 1.1 software (Bruker Daltonics, Bremen, Germany) with default settings. The smoothing, normalization, baseline subtraction and peak picking were carried out by the software, thereby creating a list of the most significant peaks of a spectrum (m/z values with a given intensity). The generated peak lists derived from the bacterial MALDI-TOF profile mass spectra were compared with each entry of the MALDI Biotyper database, which contains 3287 references, using the standard parameter of the pattern-matching algorithms. The results of pattern matching were expressed as numerical score ranging from 0 to 3.00. Following criteria were used for identification of pathogens: score ≥2.30 (2.30–3.00) secured isolate identified at species level, score 2.00 to 2.29 identified at genus level, score 1.70 to 1.99 indicates probably identified at genus and score >1.70 indicates identification was not reliable. The duplicate spot scores were recorded and highest raw scores were used for confirmation of pathogen^12^.

#### Detection of Staphylococcal species by nuc genes

All extracted bacterial DNA samples were subjected to PCR using *nuc* genes (*Saur*, *Sepi* and *Shae*) as reported in Table 2. These are universally conserved region of thermonuclease in *S*. *aureus*, *S*. *epidermidis* and *S*. *haemolyticus* located about 2 to 8 Kbp downstream of aspartate kinase gene^47–49^. Amplification was performed in 0.2 ml PCR tube in volume of 25 µL containing 1 µL of 80–100 ng of genomic DNA, 1 µL of both forward and reverse primers (20 pmoL each), 7 µL of DreamTaq Green PCR master mix (contain 0.25 mM of each dNTP, 2 mM MgCl_2_ and Taq DNA polymerase) procured from Thermo Fischer Scientific, India. The PCR was performed in a master gradient thermal cycler using following conditions: initial denaturation at 95 °C for 5 min; 30 cycles of denaturation for 30 sec at 94 °C, annealing for 30 sec at 56 °C, extension for 45 sec at 72 °C and final extension for 2 min at 72 °C followed by cooling to 4 °C until the sample was recovered. Amplified PCR products were confirmed on 1% agarose gel and image was captured in gel documentation system.

### Assessment of virulence factors

#### Coagulase activity

Isolated pure colonies (5 to10) from BPA medium were added to 1 ml of re-constituted freeze-dried rabbit plasma (HiMedia, India) to confirm inoculated pathogens are coagulase positive or negative by observing clotting of plasma after 1, 2, 4, 12 and 24 h of incubation at 37 ± 1 °C^34^.

#### Lecithinase activity

Production of lecithinase by each isolate on BPA medium containing egg yolk emulsion was evaluated based on colony appearance. Around the typical black colony, formation of precipitation zone after 48 h at 37 ± 1 °C confirmed bacterial lecithinase activity^34^.

#### Gelatinase activity

Bacterial isolates overnight grown in BPA medium were transferred to tube containing 4 mL 12% (w/v) gelatine. The tubes were incubated at 37 °C for seven days. The medium remains solid of under lack of production of geletinase. Sufficient production of gelatinase turns medium into liquid even if under refrigerated condition^34^.

#### Toxin: Hemolytic activity

Hemolytic test was performed on blood agar (BA) medium. All individual strains were streaked on BA medium and incubated at 37 °C for 24 to 48 h. After incubation, based on medium characteristics results are interpreted. Greenish zone around the colony signifies α-hemolysin; β-hemolysin (positive) and γ- hemolysin (negative) is indicated by the presence or absence of clear zone around the colonies^27^. To confirm each virulence factor tested, at least triplicate assays were performed.

#### Staphylococci characterization by mecA gene

All bacterial isolates were characterized to confirm methicillin resistance by *mecA* gene^50^ as presented in Table 2. PCR was carried out by following amplification conditions: initial denaturation at 95 °C for 4 min; 30 cycles of denaturation for 30 sec at 94 °C, annealing for 30 sec at 53 °C, extension for 1 min at 72 °C and final extension for 4 min at 72 °C followed by cooling to 4 °C until the sample was recovered. Amplified PCR product was confirmed on 1% agarose gel along with 100 bp ladder (Thermo Fischer Scientific, India) for 30 min and separated PCR products were then visualized under UV light and image was captured in gel documentation system.

#### Specific detection of MRSA

All bacterial strains were subjected to PCR amplification for specific detection of MRSA by species specific primers designed for *Coa*, *SpA*, and *Eap* genes (Table 2). PCR was carried out under following amplification conditions: initial denaturation at 95 °C for 4 min; 30 cycles of denaturation for 30 sec at 94 °C, annealing for 30 sec at 58 and 62 °C, extension for 1 min for 72 °C and final extension at 4 min at 72 °C followed by cooling to 4 °C until the sample was recovered. Amplicons were separated by subjecting 3 µl aliquots to 1% agarose gel along with 100 bp DNA ladder for 30 min. Separated PCR products were then visualized under UV light and image was captured in gel documentation system.

#### Multiplex PCR parameter

PCR amplification reaction was performed in 0.2 ml tube in volume of 25 µl containing mixture of 80–100 ng of DNA template, and 1X DreamTaq Green PCR master mix (containing 0.25 mM each of dNTP, 2 mM MgCl_2_ and Taq DNA polymerase). PCR was performed in a master gradient thermal cycler under conditions: initial denaturation at 95 °C for 5 min; 30 cycles of denaturation for 30 sec at 94 °C, annealing for 30 sec at 58 °C, extension for 45 sec at 72 °C and final extension for 2 min at 72 °C followed by cooling to 4 °C until the sample was recovered. Amplicons were separated by subjecting 3 µl aliquots to 1% agarose gel electrophoresis for 30 min along with 100 bp ladder. Separated PCR products were visualized under UV light and image was captured in gel documentation system^51^.

#### Validation of multiplex PCR (mPCR)

All primers sets used in mPCR were first validated individually with respective DNA type. If amplified product designates its predicted amplicon size, then those primer sets were used in mPCR. The *nuc* and *phoP* specific primer sets were used in mPCR reaction (Table 2). The multiplex mix was tested against all the *S*. *aureus* and *S*. *typhimurium* strains and studied as worthy only if the expected right amplicon size obtained, no false positive were observed.

#### PVL detection

LA-MRSA strains isolated were subjected for detection of specific *pvl* (*luk-PV-1* and *luk-PV-2*) gene (Table 2) encoding Panton-Valentine Leukocidin (PVL), as described by Lina *et al*.^52^.

### Microbiological confirmation of methicillin resistance and assessment of MDR antimicrobial resistance pattern

#### Antibiotic agar dilution method

All *Staphylococci* strains were subjected to agar dilution method according to Ghanwate *et al*.^17^, method to assess the antibiotic susceptibility. Bacterial suspension was prepared from overnight culture and inoculated on to nutrient agar containing 10 μL of different serial dilutions of antibiotics. Control was maintained without any antibiotic and incubated at 37 °C for 24 h. After overnight incubation, any specific concentration that inhibited growth indicated the MIC for that particular strain and isolates were categorized as susceptible or resistant to antibiotics as per Clinical & Laboratory Standards Institute (CLSI). Assays were performed in triplicates for all strains.

#### Antibiotic MDR susceptibility testing for MRSA

Resistance to antibiotics was confirmed as per the guidelines of internationally recognized standards of the Clinical and Laboratory Standards Institute (CLSI)^53^. MRSA, MRSE and MRSH strains were screened for susceptibility by 22 antimicrobial agents using disc diffusion method on Muller-Hinton agar. The antibiotics disc tested were (concentration in μg, HiMedia, India) Erythromycin (E-15 μg), Clindamycin (CD-2 μg), Gentamicin (GEN-10 μg), Cefotaxime (CTX-30 μg), Norfloxacin (NX-10 μg), Ciprofloxacin (CIP-5 μg), Tetracycline (TE-30 μg), Rifampicin (RIF-5 μg), Nitrofurantoin (NIT-300 μg), Linezolid (LZ-30 μg), Chloramphenicol (C-30 μg), Amoxyclav (AMC-10 μg), Amphicillin (AMP-10 μg), Azithromycin (AZM-15 μg), Bacitracin (B-10 Units), Cefoxitime (CFM-5 μg), co-Trimoxazole (COT-23.75 μg), Kanamycin (K- 30 μg), Oxacillin (OX-I μg), Penicillin G (P-10 Units), Streptomycin (S-100 μg), Tetracycline (TE-30 μg) and Tobramycin (TOB-30 μg). The results were recorded after 24 h of incubation at 37 °C and interpreted as per CLSI guidelines. Screened isolates showing resistance to more than three classes of non-β-lactam antibiotics were considered as multidrug resistant (MDR) strains, as described by Chajecka *et al*.,^9^. Assay was performed in triplicates for all strains.

#### Micro dilution-based method

Assay was performed in 96 well plates containing 100 μL of bacterial broth culture, 10 μL of varying concentrations of different antibiotics in each well according to Ghanwate *et al*.^17^ and plates were incubated for 6 h at 37 °C. After incubation, 20 μL of 10 mg/mL triphenyl tetrazolium chloride (TTC) in phosphate buffer was added to each well and incubated for 1 h. Formation of blue color is the indication for viable bacteria and visual MIC break point was determined for respective drug concentration. Assay was performed in triplicates for all strains.

### Polymerase Chain Reaction-Single Strand Conformation Polymorphism (PCR-SSCP) studies

#### Sample preparation

Diverse bacterial DNA was subjected to PCR for amplification of conserved universal 16 S rRNA region. Amplified PCR product (400 ng/25 μL) was mixed with 25 μL denaturing buffer (95% formamide, 20 mM ethylenediaminetetraacetic acid (EDTA) and 0.05% bromophenol blue). The mixture was denatured at 95 °C for 5 min and chilled for 10 min on ice^54^.

#### Non-denaturing polyacrylamide gel electrophoresis

The denatured (9 μL) PCR products and 10 μL of 25 ng ssDNA ladder were loaded on 8% acrylamide-bisacrylamide non denaturing gel [8 ml contained: 4.75 ml H_2_O, 2 ml 10X TBE, 1.2 ml 2X acrylamide-bisacrylamide, 40 μl of 10% ammonium persulphate (APS) and 16 μL TEMED] and electrophoresis was done at 150 V, 240 mA and 50 W in prechilled 1X TBE tank buffer for 2 h at room temperature^54^.

#### Silver staining

After electrophoresis, gel was removed from the unit and silver stained as per the protocol described by Kong *et al*.,^54^; gel was soaked in 50 ml of 10% ethanol for 10 min, and placed with the similar amount of 1% nitric acid for 3 min. After two brief washes with 100 mL distilled water, gels were stained with 50 mL of 2 ppm silver nitrate (made from 100X stock stored at 4 °C) for 20 min followed by three times rinse with 200 mL of distilled water. Gels were developed by brief rinsing in 30 mL of 1 ppm formaldehyde in 3% sodium carbonate until desired band intensity was reached. Reaction was stopped using 1% acetic acid once the SSCP patterns were visible and image pattern was captured and documented.

### Statistical analysis

All studies were implemented in triplicate. All data are expressed as the mean ± standard deviation (SD) and P values of less than 0.05 were considered statistically significant.

## Electronic supplementary material

MALDI-TOF-MS based identification and molecular characterization of food associated methicillin-resistant Staphylococcus aureus
