# Supplementary material for: MALDI-TOF-MS based identification and molecular characterization of food associated methicillin-resistant Staphylococcus aureus
Source: Sci Rep. 2017 Sep 12;7:11414. doi: 10.1038/s41598-017-11597-z (PMC5595867; doi:10.1038/s41598-017-11597-z)
Supplement: Supplementary file 1 — MALDI-TOF-MS based identification and molecular characterization of food associated methicillin-resistant Staphylococcus aureus [file 41598_2017_11597_MOESM1_ESM.pdf]

Supplementary Information

MALDI-TOF-MS based identification and molecular characterization of food associated methicillin-resistant *Staphylococcus aureus*

Manukumar, H. M. and Umesha, S*.

Department of Studies in Biotechnology, University of Mysore, Manasagangotri, Mysuru-570006, Karnataka, INDIA.

***Corresponding author**

E-mail: pmumesh@gmail.com, su@appbot.uni-mysore.ac.in

Phone: 91 0821 2419884; 09449264384

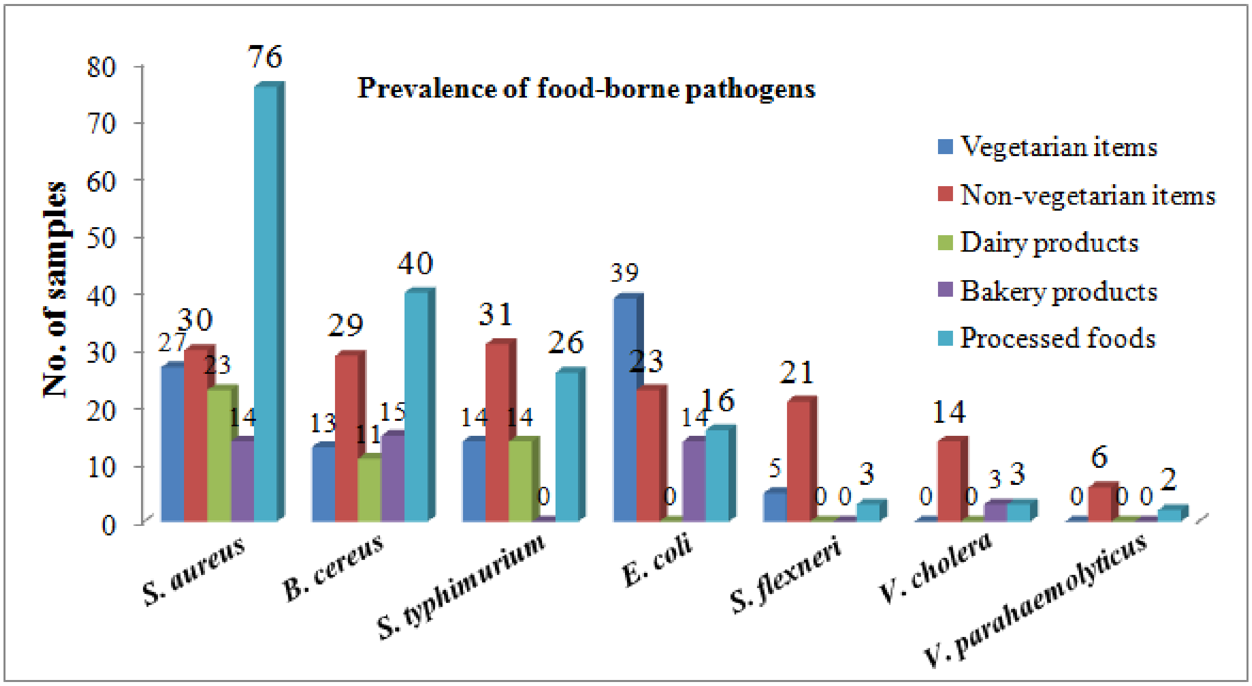

Supplementary Fig S1. Different food samples and associated food-borne pathogens. Varieties food samples collected from different regions of Mysuru, India and isolated diverse pathogens from food samples associated.

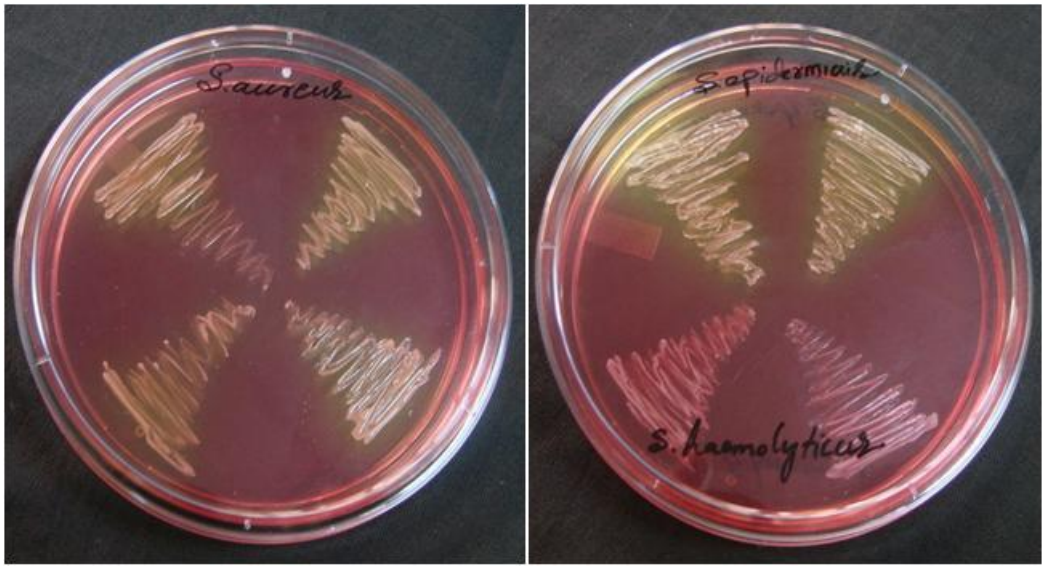

A.

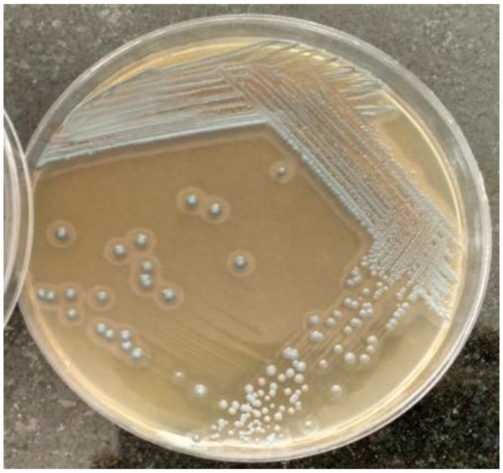

B.

Supplementary Fig S2. Food-borne *Staphylococcal* spp. on selective medium. **A.** This figure indicating the *S. aureus, S. epidermidis* and *S. haemolyticus* pathogens on selective Mannitol Salt Phenol-red Agar medium. **B.** Indicating the *S. aureus* on Baired Parker Agar medium

E. coli

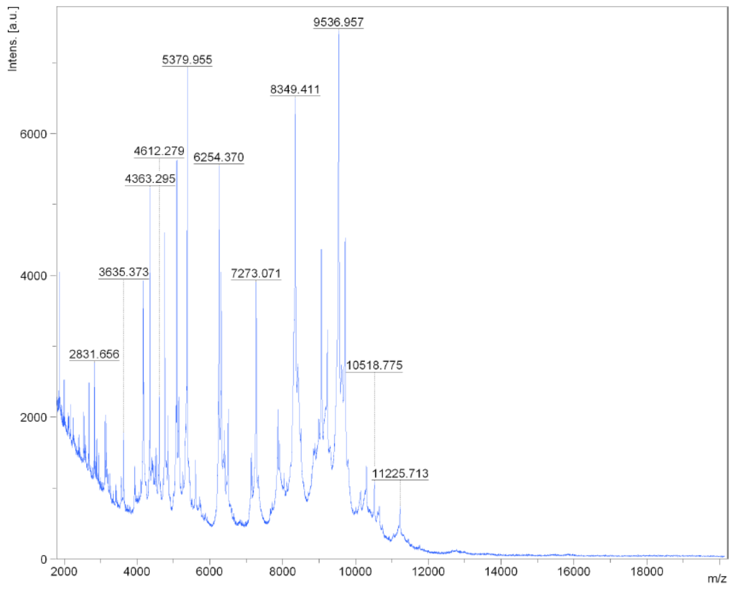

B. cereus

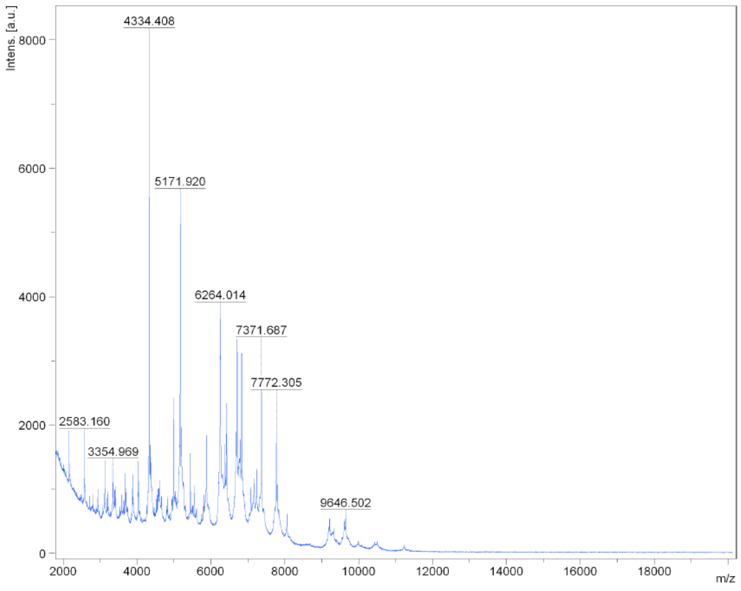

S. typhimurium

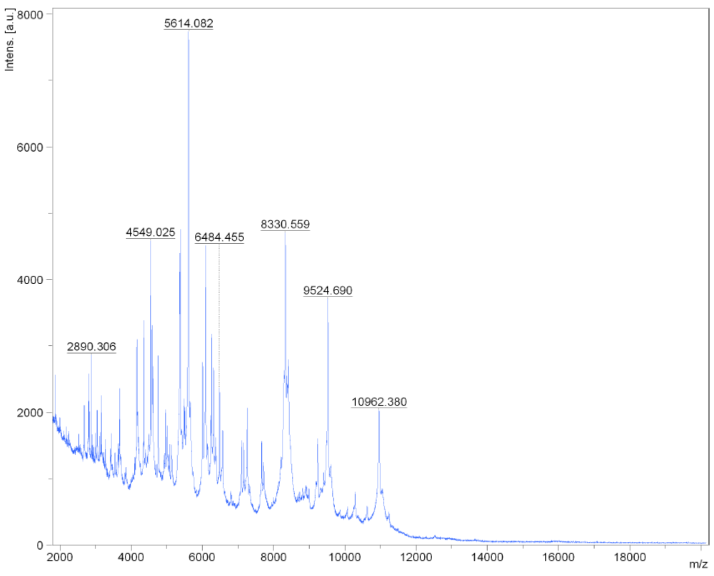

S. flexneri

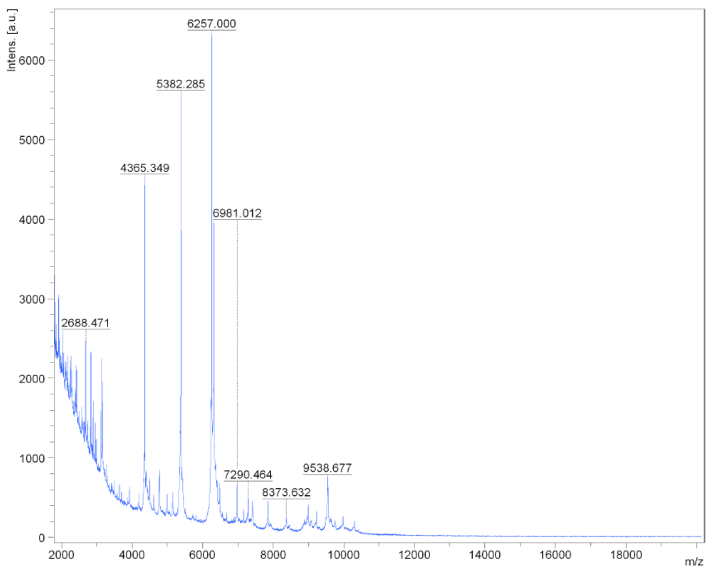

P. aeruginosa

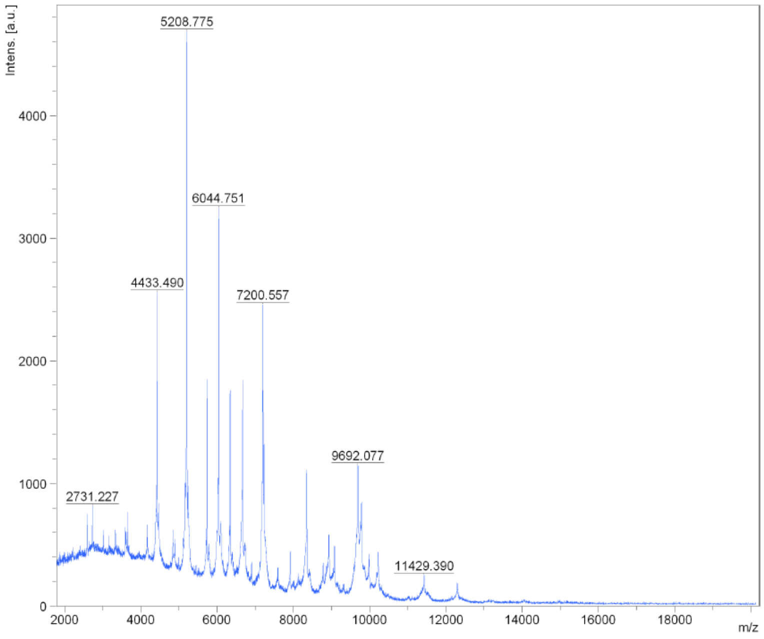

V. cholera

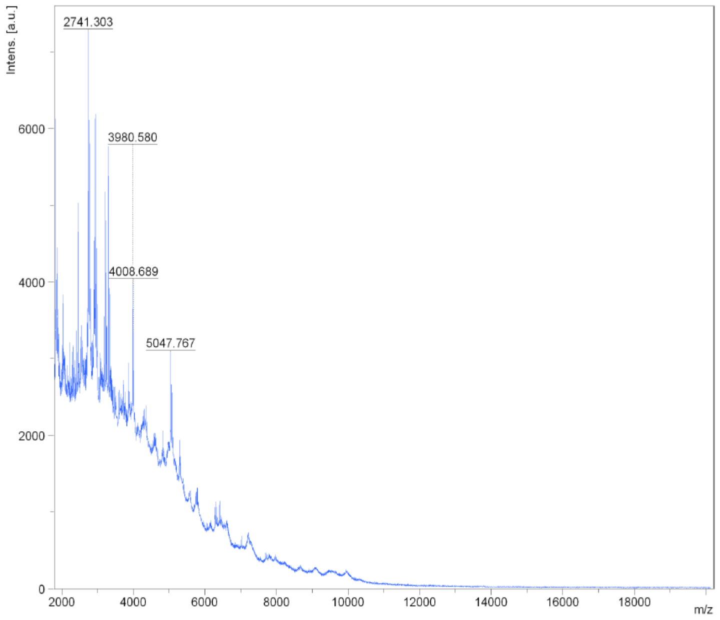

V. parahaemolyticus

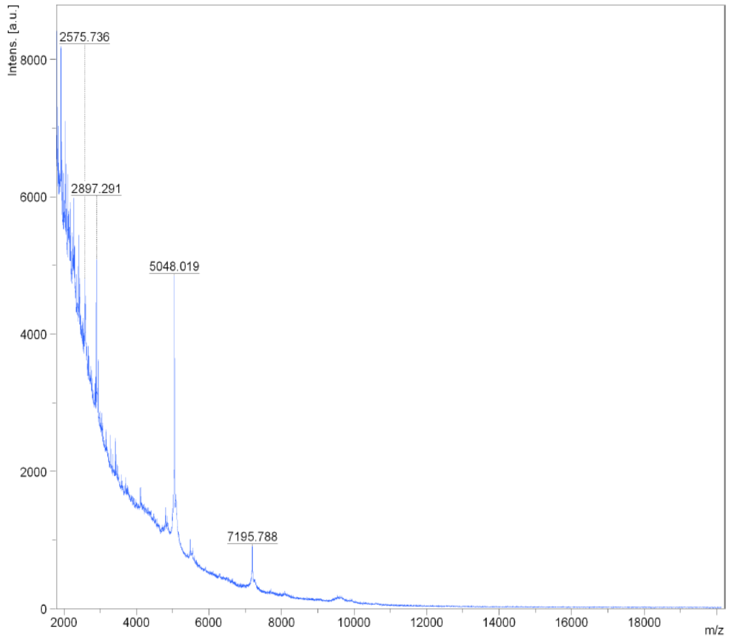

E. aerogenes

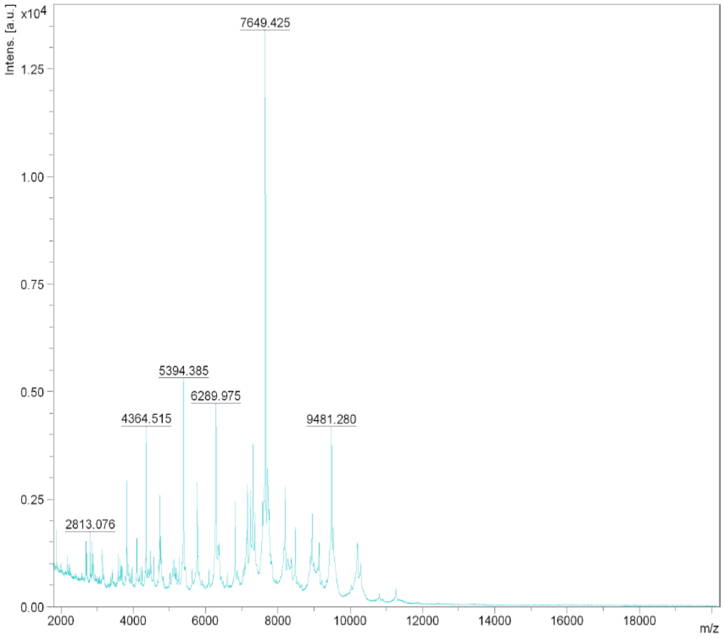

Supplementary Fig S3. Different food-borne pathogens MALDI-TOF-MS spectra.

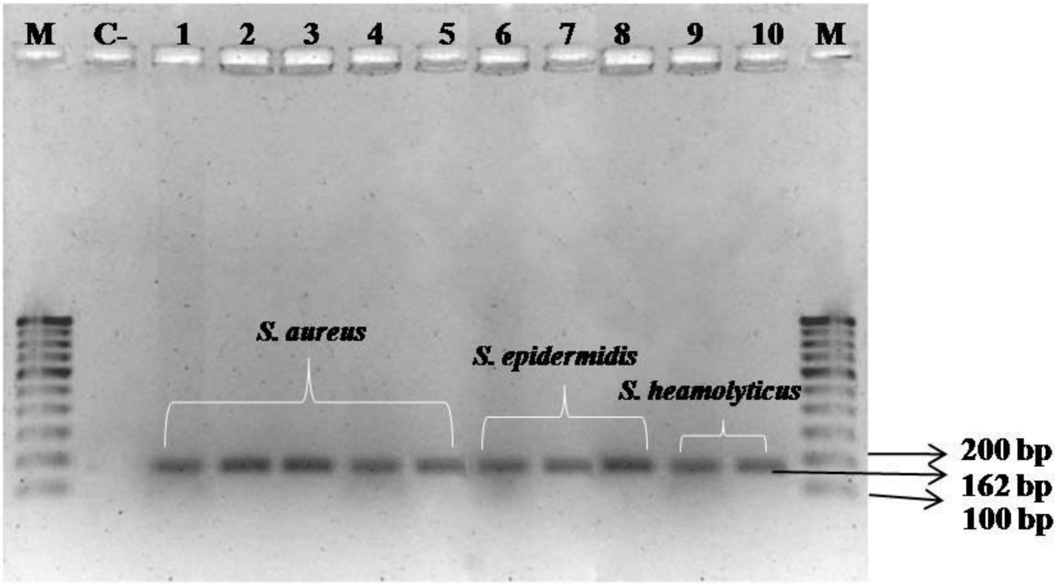

Supplementary Fig S4. Multi drug resistant (MDR) ***Staphylococcal*** **isolates conserved** ***mecA*** **gene.** All the *S. aureus* (1-5), *S. epidermidis* (6-8) and *S. haemolyticus* (9 and 10) amplified by *mecA* gene and amplified *mecA* gene product of 163 bp on agarose gel, along with 100 bp ladder (M) with negative control (C-).

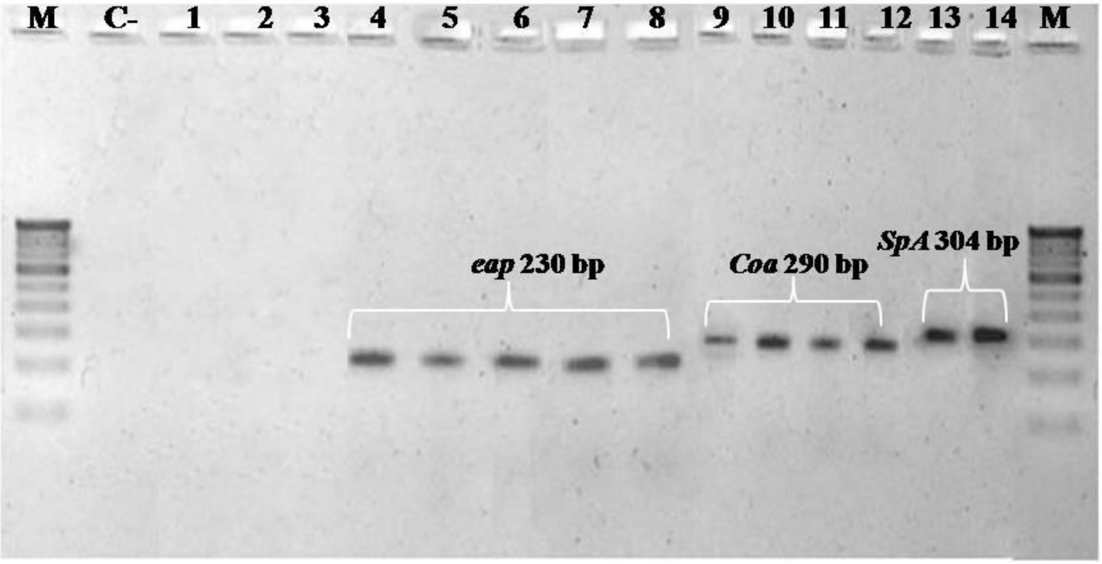

Supplementary Fig S5. The specific identification of *Staphylococcus aureus* was assessed by species specific primers *Eap* (Lane 4-8), *Coa* (Lane 9-12) and *SpA* (Lane 13&14) genes and amplified product confirmed 230, 290 and 304 bp size on agarose gel electrophoresis respectively. Lane 1-3 *E. coli, S. typhimurium,* and *S. flexneri* respectively; C- indicates negative control and Lane M represent 100 bp ladder.

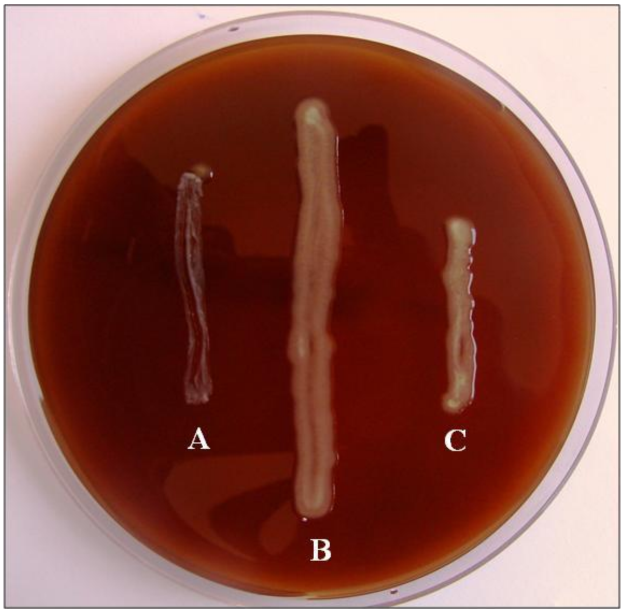

Supplementary Fig S6. Hemolytic activity of *Staphylococcal* species. Blood Agar medium exhibiting hemolytic activity: *S. haemolyticus* (A) showing γ-hemolytic, *S. epidermidis* (B) and *S. aureus* (C) shows α -hemolytic property.

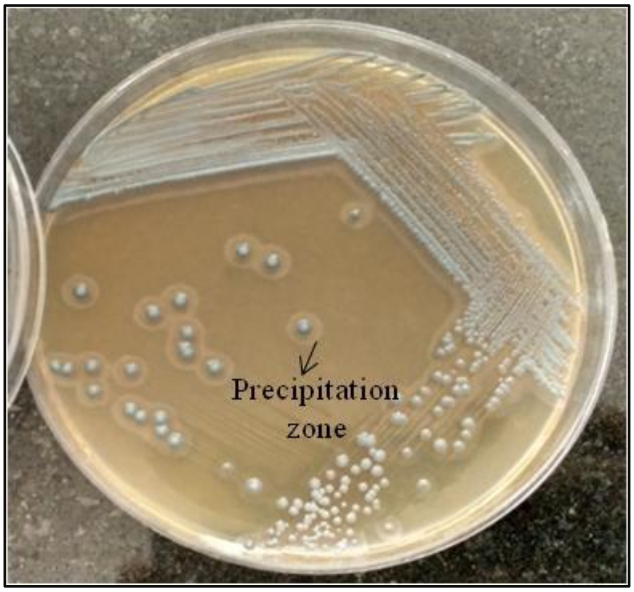

Supplementary Fig S7. Lecithinase activity of *Staphylococcus aureus* on BPA medium.

Supplementary Table S1. Different vegetarian samples, locations and specific pathogen isolation.

Sl. No.	Samples	Location	Total No. of samples collected	Observations						
				***Staphylococcal* spp.**	***B. cereus***	***S. typhimurium***	***E. coli***	***S. flexneri***	***V. cholera***	***V. parahemolyticus***
1	Carrot	A, B, E	16	9	1	0	9	0	0	0
2	Beans	A, B	8	4	0	0	4	0	0	0
3	Capsicum	A, E	2	0	1	2	1	0	0	0
4	Cauliflower	C	2	1	0	0	1	0	0	0
5	Brinjal	A, B, D	10	2	2	0	5	0	0	0
6	Cabbage	A, C, E	3	1	1	0	1	0	0	0
7	Tomato	A, B, C	15	2	2	0	0	2	0	0
8	Radish	A, D	3	0	0	0	0	0	0	0
9	Ridge gourd	A, D	4	1	0	0	0	0	0	0
10	Bitter gourd	A, B	5	0	0	0	0	0	0	0
11	Cucumber	A, E	3	0	0	0	0	0	0	0
12	Chilli	A, B, C	9	1	2	1	2	0	0	0
13	Beet root	A, E	3	0	0	0	0	0	0	0
14	Ladies finger	A, B, D	4	0	0	1	1	0	0	0
15	Onion	A, B, C	13	0	1	0	5	3	0	0
16	Potato	B, C	8	2	1	0	2	0	0	0
17	Amaranth	A, C	2	0	0	0	0	0	0	0
18	Apple	A, C	7	3	1	0	2	0	0	0
19	Pineapple	B, C, E	3	1	0	0	1	0	0	0
20	Banana	A, B	4	0	0	0	1	0	0	0
21	Grapes	A, B	4	0	0	0	1	0	0	0
22	Orange	A, B	7	0	1	0	2	0	0	0
23	Gooseberry	C, E	2	0	0	0	1	0	0	0
**Total**			**137**	**27**	**13**	**4**	**39**	**5**	**0**	**0**

Supplementary Table S2. Different Non-vegetarian samples, locations and specific pathogen isolation.

Sl. No	Samples	Location	No. of samples collected	Observation						
				***Staphylococcal* spp.**	***B. cereus***	***S. typhimurium***	***E. coli***	***S. flexineri***	***V. cholera***	***V. parahemolyticus***
1	Chicken	A,C, D	20	6	4	3	5	2	2	1
2	Mutton	A, B, C	15	4	8	2	4	3	3	0
3	Fish	A, D, E	14	7	5	4	6	5	1	0
4	Crab	D	4	3	4	6	3	3	5	2
5	Prawn	D	4	2	3	7	2	3	3	1
6	Pork	D, E	5	3	2	5	2	4	0	2
7	Beef	D	7	5	3	4	1	1	0	0
**Total**			**69**	**30**	**29**	**31**	**23**	**21**	**14**	**6**

Supplementary Table S3. Different dairy samples, locations and specific pathogen isolation.

Sl. No	Samples	Location	No. of samples collected	Observation						
				Staphylococcal spp.	*B. cereus*	*S. typhimurium*	*E. coli*	*S. flexineri*	*V. cholera*	*V. parahemolyticus*
1	Milk	A,B, C, D	65	16	5	2	0	0	0	0
2	Curd	A, D, E	4	2	3	0	0	0	0	0
3	Ice cream	A,G	8	1	2	0	0	0	0	0
4	Ghee	A,B	5	1	1	0	0	0	0	0
5	Paneer	A,C, D	5	3	0	2	0	0	0	0
**Total**			**87**	**23**	**11**	**4**	**0**	**0**	**0**	**0**

Supplementary Table S4. Different bakery samples, locations and specific pathogen isolation.

**Sl. No**	**Samples**	**Location**	**No. of samples collected**	**Observation**						
				***Staphylococcal* spp.**	***B. cereus***	***S. typhimurium***	***E. coli***	***S. flexineri***	***V. cholera***	***V. parahemolyticus***
1	Cake	A,B	6	2	1	0	1	0	0	0
2	Mysore pak	A,B, D	6	1	1	0	1	0	0	0
3	Bread	A,B	7	2	2	0	0	0	0	0
4	Rusk	A,B	8	0	2	0	2	0	0	0
5	Jamun	A, B, C	3	0	0	0	1	0	0	0
6	Rasgulla	A,D	8	0	0	0	0	0	0	0
7	Chips	A,B	16	3	4	0	4	0	0	0
8	Fried ground nut	A,D	4	4	3	0	2	0	2	0
9	Fried dhal	A, C, E	5	2	2	0	2	0	1	0
10	Saunpapdi	A, B, E	8	0	0	0	1	0	0	0
11	Biscuit	A	1	0	0	0	0	0	0	0
**Total**			**72**	**14**	**15**	**0**	**14**	**0**	**3**	**0**

Supplementary Table S5. Different processed samples, locations and specific pathogen isolation.

Sl. No	Samples	Location	No. of samples collected	Observation						
				***Staphylococcal* spp.**	***B. cereus***	***S. typhimurium***	***E. coli***	***S. flexineri***	***V. cholera***	***V. parahemolyticus***
1	Color rice	A, D, E	34	14	10	1	3	0	0	0
2	Idly	A, D	43	12	3	0	1	0	0	0
3	Chapathi	A, B	8	9	2	2	2	0	0	0
4	Chicken	C, D, E	16	12	8	9	3	1	0	0
5	Mutton	A, B	5	3	5	1	2	0	0	2
6	Kabab	A,A	15	13	9	8	1	2	3	0
7	Paulav	A	3	0	1	2	1	0	0	0
8	Sambar	A	4	1	1	2	2	0	0	0
9	Vada	A,C	5	1	1	1	1	0	0	0
10	Egg	A,B	2	1	0	0	0	0	0	0
**Total**			**135**	**66**	**40**	**26**	**16**	**3**	**3**	**2**

A: Devaraj Urs market, Mysore, B: T. K. Layout, Mysore, C: K. D. Road, Mysore, D: Bamboo bazaar, Mysore, and E: Nanjanagud.

Supplementary Table S6. MDR *Staphylococcal* species associated with different food items.

**Food items**	**Areas/Source**	***S. aureus***	***S. epidermidis***	***S. haemolyticus***
Vegetarian	Hospital	22	4	1
	Street vended	54	1	0
	Bakery	14	0	0
Non-vegetarian	Market	30	1	2
	Street vended	12	1	1
	Packed	6	1	0
Dairy	Milk	12	0	0
	Milk products	11	1	0
Vegetables and fruits	Market	27	0	0
	City	8	0	0

Supplementary Table S7. Different pathogens and confirmed at species level.

Sl. No.	Microorganisms	Standard	Isolated isolates		% of incidence	Specific genes							
			**Total**	**Confirmed**		***Eap***	***Emitic toxin***	***phoP***	***stx***	***mpl***	***Scaffolding protein***	***ompW***	***toxR***
1	*Staphylococcus aureus*	1	160	152	94.37	+	-	-	-	-	-	-	-
2	*Bacillus cereus*	1	108	97	89.81	-	+	-	-	-	-	-	-
3	*Salmonella typhimurium*	1	85	60	70.58	-	-	+	-	-	-	-	-
4	*Escherichia coli*	1	92	78	84.78	-	-	-	+	-	-	-	-
5	*Shigella flexneri*	1	29	20	68.96	-	-	-	-	-	+	-	-
6	*Vibrio cholera*	1	20	8	40.00	-	-	-	-	-	-	+	-
7	*Vibrio parahemolyticus*	1	8	2	25.00	-	-	-	-	-	-	-	+
8	*Pseudomonas aeruginosa*	1				-	-	-	-	-	-	-	-
9	*Bacillus subtilis*	1				-	-	-	-	-	-	-	-
10	*Enterobacter aerogenes*	1				-	-	-	-	-	-	-	-

Supplementary Table S8: Biochemical characterization of food-borne *Staphylococcal* isolates.

**Biochemical tests** [a]	**Results**		
	**Sa**	**Se**	**Sh**
Gram's reaction	+	+	+
Catalase test	+	+	+
Kovac's oxidase	-	-	-
KOH solubility	+	+	+
Starch hydrolysis	+	-	-
Gelatin hydrolysis	+	w	w
Coagulase	+	-	-
Lipase	+	-	+
DNase	+	-	-
Pigment	+	-	-

Sa: *S. aureus,* Se: *S. epidermidis* and Sh: *S. haemolyticus.* [a]All the test results are performed in five replicates and were repeated thrice. '+' indicates positive reaction, '-' indicates negative reaction and 'w' indicates weak reaction.

Supplementary Table S9: DNA sequence results of pathogens involved in this study.

Sl. No.	Pathogen	Strain	Gene	GenBank Accession no
1	*S. aureus*	UOM012	*16S rRNA*	KX641603
2	*S. aureus*	UOM018	*16S rRNA*	KX431901
3	*S. aureus*	UOM048	*16S rRNA*	KX431902
4	*S. aureus*	UOM051	*16S rRNA*	KX431903
5	*S. aureus*	UOM069	*16S rRNA*	KX641604
6	*S. aureus*	UOM082	*16S rRNA*	KX431904
7	*S. aureus*	UOM090	*16S rRNA*	KX355577
8	*S. aureus*	UOM114	*16S rRNA*	KX431905
9	*S. aureus*	UOM136	*16S rRNA*	KX641605
10	*S. aureus*	UOM157	*16S rRNA*	KX431906
11	*S. aureus*	UOM160	*16S rRNA*	KX431907
12	*S. aureus*	UOM090	*mecA*	KX371863
13	*S. epidermidis*	UOM036	*16S rRNA*	KX371861
14	*S. epidermidis*	UOM057	*16S rRNA*	KX431908
15	*S. epidermidis*	UOM080	*16S rRNA*	KX641606
16	*S. epidermidis*	UOM097	*16S rRNA*	KX431909
17	*S. epidermidis*	UOM036	*mecA*	KX387571
18	*S. haemolyticus*	UOM142	*16S rRNA*	KX371862
19	*S. haemolyticus*	UOM150	*16S rRNA*	KX431910
20	*S. haemolyticus*	UOM142	*mecA*	KX387572
21	*V. cholera*	UOM210	*16S rRNA*	KX452112
22	*V. parahaemolyticus*	UOM300	*16S rRNA*	KX452113
23	*E. coli*	UOM352	*16S rRNA*	KX452114
24	*S. flexneri*	UOM398	*16S rRNA*	KX452115
25	*S. typhimurium*	UOM400	*16S rRNA*	KX452116
26	*E. aerogenes*	UOM447	*16S rRNA*	KX452117
27	*B. cereus*	UOM380	*16S rRNA*	KX641607
28	*P. aeruginosa*	UOM219	*16S rRNA*	KX641608

Supplementary Table S10: Comparison of methods involved in pathogen identification.

	Standard culture	PCR	Sequencing	PCR-SSCP	MALDI-Biotyper
**Assay time (sample processing to detection)**	2-7 days	> 3 hr	Within a day	< 5 hr	< 10-30 min
**Instrument**	Incubator, Biosafety Laminar hood Rs. 50,000-2,00,000	Standard PCR machine Rs. 3,00,000 – 4,00,000	Automated sequencer Rs. 7,00,000	Electrophoresis unit Rs.25,000	Bruker Daltonics MALDI-TOF-MS ~Rs. 1,20,00,000
**Cost (per assay)**	~ Rs. 50 (media, plates, chemicals)	~ Rs. 60 (polymerases, dyes, primers, agarose)	~ Rs. 500 (PCR, purification, chemicals)	~ Rs. 90 (chemicals)	~ Rs. 2,500 (chemicals and instrumentation)
**POC use**	Difficult to apply	Easily applicable	Difficult to use	Easily applicable in the future	Might be applicable in the future, currently using in facilitated diagnostic labs
**Care**	Given at highest priority	Maintain hygiene	Maintain hygiene	Care should be taken	Work with sterile condition
**Sensitivity**	>80-85%	>90-94%	>90-96%	>95-97%%	>99%

Supplementary Table S11: Reference identification of pathogenic isolates obtained by MALDI-Biotyper and biochemical assays.

Sl.No.	Original biochemical identification	Biotyper ID (score)	Repeated biochemical identification	16S rDNA identification
1	*S. aureus*	*S. aureus* (2.28)	*S. aureus*	*S. aureus*
2	*S. epidermidis*	*S. epidermidis* (2.10)	*S. epidermidis*	*S. epidermidis*
3	*S. haemolyticus*	*S. haemolyticus* (2.01)	*S. haemolyticus*	*S. haemolyticus*
4	*S. typhimurium*	*S. typhimurium* (2.18)	*S. typhimurium*	*S. typhimurium*
5	*S. flexneri*	*S. flexneri* (2.03)	*S. flexneri*	*S. flexneri*
6	*E. coli*	*E. coli* (2.32)	*E. coli*	*E. coli*
7	*B. cereus*	*B. cereus* (2.00)	*B. cereus*	*B. cereus*
8	*P. aeruginosa*	*P. aeruginosa* (2.10)	*P. aeruginosa*	*P. aeruginosa*
9	*V. cholera*	*V. cholera* (2.01)	*V. cholera*	*V. cholera*
10	*V. parahaemolyticus*	*V. parahaemolyticus* (2.00)	*V. parahaemolyticus*	*V. parahaemolyticus*
11	*E. coli*	*E. aerogenes* (2.44)	*E. coli*	*E. aerogenes*

Supplementary Table S12: Molecular mechanism of antibacterial resistance identified in CoPS.

Original biochemical test identification		Phenotype	Resistant gene	Food source
S. aureus	**UOM012**	*AMP, AMC, CTX, OX, P, COT,TE*	*mecA*	*Tomato, Onion, Brinjal*
S. aureus	**UOM018**	*LZ, CIP, K, OX, P, C, CFM, B*	*mecA*	*Vada, Paulav*
S. aureus	**UOM048**	*AZM, B, CFM, E, OX, P, RIF, S*	*mecA*	*Colour rice,*
S. aureus	**UOM051**	*TE, NIT, K, TE, AZM*	*mecA*	*Chips,*
S. aureus	**UOM069**	*E, AMC*	*mecA*	*Chips, Milk*
S. aureus	**UOM082**	*S, TE, NIT, AMP, K, TOB, OX, P, CIP, C, AMC, AZM, B, CFM*	*mecA*	*Fish kabab, Chicken*
S. aureus	**UOM090**	*CD, NIT, K, TE, AZM, B, NX, CTX, P, OX, RIF, S, C, TOB*	*mecA*	*Idli, Vada, Paulav, Mutton, Chicken*
S. aureus	**UOM114**	*NIT, AMP, TOB, RIF,COT, OX, P, C, CFM, B*	*mecA*	
S. aureus	**UOM136**	*TOB, RIF,COT, AMP, B, K, OX, P*	*mecA*	*Apple, Bread*
S. aureus	**UOM157**	*TOB, CD, CFM*	*mecA*	*Colour rice, Paulav, Vada*
S. aureus	**UOM160**	*TE, OX, P, AMC*	-	*Grapes, Apple*
S. epidermidis	**UOM036**	*S, TE, B, CFM, OX, K, P, RIF, S, AMC, CTX*	*mecA*	*White rice (Street vender)*
S. epidermidis	**UOM057**	*S, TE, NIT, K, AMP, TOB, OX,COT, CIP, C, AMC, AZM, B, CFM*	*mecA*	*Soya milk Idli*
S. epidermidis	**UOM080**	*NIT, AMP, TOB, CFM, E, OX, S, TE*	*mecA*	
S. epidermidis	**UOM097**	*TE, AZM, RIF,COT, AMP, B, CFM*	*mecA*	*Carrot, Beet root*
S. haemolyticus	**UOM142**	*NIT, TE, AZM, K, B, CFM, CTX, AMP, TOB, CFM, E, OX*	*mecA*	*Pork, Fish, Mutton Chicken, Crab, Idli*
S. haemolyticus	**UOM150**	*TOB, COT, C, K, NIT, TE, AZM, E, AMC, OX, P*	*mecA*	*Idli, Tomato*

Abbreviations: AMP- amphicilin, AZM-azithromycin, AMC- amoxyclav, B- bacitracin, CFM- cefoxitin, CTX- cefotaxime, C- chloramphenicol, CIP- ciprofloxacin, CD- clindamycin, COT- co-Trimoxazole, E-erythromycin, GEN- gentamycin, K- kanamycin, LZ- linezolid, NIT- nitrofurantoin, NX- norfloxin, OX-oxacillin, P- pennicilin, RIF- rifampicin, S- streptomycin, TE- tetracycline, and TOB- tobramycin.

Supplementary Table S13: Proposed resistance genes conferred in our *Staphylococcal* species.

Drugs	Gene conferring resistance	Description
**Amoxyclav**	*amc*	Inhibiting bacterial cell wall synthesis
**Amphicillin**	*amr*	Inhibits the third and last stage of bacterial cell wall synthesis by binding to PBPs. Cell lysis is then mediated by bacterial cell wall autolytic enzymes such as autolysins; it is possible that Ampicillin interferes with an autolysin inhibitor
**Azithromycin**	*ermC*	Binds to the 23S rRNA of 50S subunit of the 70S bacterial ribosomes, and therefore inhibits RNA-dependent protein synthesis in bacterial cells
**Bacitracin**	*baca*	Undecaprenyl pyrophosphate phosphatase, which consists in the sequestration of Undecaprenyl pyrophosphate
**Cefotaxime**	*mecA*	Inhibition of cell wall synthesis via affinity for penicillin-binding proteins (PBPs). Cefotaxime shows high affinity for penicillin-binding proteins in the cell wall including PBP Ib and PBP III
**Cefoxitime**	*mecA*	Inhibition of cell wall synthesis
**Chloramphenicol**	cml_e4	Major facilitator superfamily transporter, chloramphenicol efflux pump
**Ciprofloxacin**	*norA*	Inhibition of the enzymes topoisomerase II (DNA gyrase) and topoisomerase IV, which are required for bacterial DNA replication, transcription, repair, strand supercoiling repair, and recombination.
**Clindamycin**<br>**Clindamycin (inducible)**	*VGA(A)LC*<br>*ermA/ermB,*	Bind to the 23S portion of the 50S subunit of bacterial ribosomes and inhibit early elongation of peptide chain by inhibiting transpeptidase reaction
**Co-Trimoxazole**	*dhfr*	Inhibits bacterial synthesis of dihydrofolic acid and inhibiting the enzyme dihydrofolate reductase by competing with PABA. This combination blocks 2 consecutive steps in bacterial biosynthesis of essential nucleic acids and proteins
**Erythromycin**	*msrA*<br>*ermC/ermT*	Inhibits protein synthesis by reversibly binding to 50S ribosomal subunits of ribosomenear the ‘P’ site to stop the translation. Effective against actively dividing organisms.
**Gentamicin**	*aacA-aphD*	Works by irreversibly binding the 30S subunit of the bacterial ribosome, interrupting protein

		synthesis similar to Kannamycin
**Kanamycin**	*aacA-aphD*	Binds irreversibly to the bacterial 30S ribosomal subunit protein and 16S rRNA and prevents the formation of the initiation complex with messenger RNA intern inhibits bacterial protein synthesis
**Linezolid**	*Cfr*	Bacteriostatic action, which stops bacteria from reproducing
**Methicillin**	*mecA*	Penicillin binding protein, which has a low affinity for beta-lactams and catalyze a penicillin-insensitive transpeptidation
**Nitrofurantoin**		Damage bacterial DNA
**Norfloxacin**	*nfxC*	Extrusion of norfloxacin
**Oxacillin**	*mecA*	Binding to specific penicillin-binding proteins (PBPs) located inside the bacterial cell wall. Inhibits the third and last stage of bacterial cell wall synthesis
**Pennicillin G**	*blaZ/* bl2a_pc	Class A beta-lactamase. This enzyme breaks the beta-lactam antibiotic ring open and deactivates the molecule's antibacterial properties
**Rifampicin**	*Rif*	RNA polymerase with reduced affinity
**Streptomycin**	Str	Streptomycin resistance protein
**Tetracycline**	*tetK/tetL/tetM*	Major facilitator superfamily transporter, tetracycline efflux pump
**Tobramycin**	aac6ie/ *aacA-aphD*	Aminoglycoside N-acetyltransferase, which modifies aminoglycosides by acetylation.
